# Supplementary material for: Prevalence and changing antimicrobial resistance profiles of Shigella spp. isolated from diarrheal patients in Kolkata during 2011–2019
Source: PLoS Negl Trop Dis. 2024 Feb 20;18(2):e0011964. doi: 10.1371/journal.pntd.0011964 (PMC10906866; doi:10.1371/journal.pntd.0011964)
Supplement: S1 Table — (DOCX) [file pntd.0011964.s001.docx]

**S1 Table**

| ***Target*** | **Primer** | **Sequence (5’-3’)** | **Annealing**  **Temperature (°C)** | **Base pair** | **Reference** |
| --- | --- | --- | --- | --- | --- |
| *ipaH* | *ipaH_F* | GTTCCTTGACCGCCTTTCCGATACCGTC | 60 | 620 | [1] |
|  | *ipaH_R* | GCCGGTCAGCCACCCTCTGAGAGTAC |  |  |  |
| *ial* | *ial_F* | CTGGATGGTATGGTGAGG | 55 | 320 | [1] |
|  | *ial_R* | GGAGGCCAACAATTATTTCC |  |  |  |
| *set* | *set_F* | TCACGCTACCATCAAAGA | 54 | 309 | [1] |
|  | *set_R* | TATCCCCCTTTGGTGGTA |  |  |  |
| *sen* | *sen_F* | ATGTGCCTGCTATTATTTAT | 55 | 799 | [1] |
|  | *sen_R* | CATAATAATAAGCGGTCAGC |  |  |  |
| *virF* | *virF_F* | CCTCAGAATAGGAGTGTTG | 55 | 493 | [2] |
|  | *virF_R* | AGCTGCATAAGCTCTTTCTTC |  |  |  |
| *virB* | *virB_F* | CGATAGATGGCGAGAAATTATATCCCG | 56 | 766 | [3] |
|  | *virB_R* | CGATCAAGAATCCCTAACAGAAGAATCAC |  |  |  |
| *sat* | *sat_F* | TCAGAAGCTCAGCGAATCATTG | 59 | 930 | [4] |
|  | *sat_R* | CCATTATCACCAGTAAAACGCACC |  |  |  |
| *sigA* | *sigA_F* | CCGACTTCTCACTTTCTCCCG | 58 | 430 | [4] |
|  | *sigA_R* | CCATCCAGCTGCATAGTGTTTG |  |  |  |
| *sep* | *sep_F* | GCAGTGGAAATATGATGCGGC | 58 | 794 | [4] |
|  | *sep_R* | TTGTTCAGATCGGAGAAGAACG |  |  |  |
| *pic* | *pic_F* | ACTGGATCTTAAGGCTCAGGAT | 58 | 572 | [4] |
|  | *pic_R* | GACTTAATGTCACTGTTCAGCG |  |  |  |

**Reference:**

1. Casabonne C, González A, Aquili V, Balagué C. Prevalence and Virulence Genes of Shigella spp. Isolated from Patients with Diarrhea in Rosario, Argentina. Jpn J Infect Dis. 2016;69: 477–481. doi:10.7883/yoken.JJID.2015.459

2. Wang S-J, Chen JH. A rapid and specific PCR method for the detection of Shigella spp. in spiked samples. Journal of Food and Drug Analysis. 2020;20. doi:10.38212/2224-6614.2081

3. Fan W, Qian H, Shang W, Ying C, Zhang X, Cheng S, et al. Low distribution of genes encoding virulence factors in Shigella flexneri serotypes 1b clinical isolates from eastern Chinese populations. Gut Pathogens. 2017;9: 76. doi:10.1186/s13099-017-0222-9

4. Moosavian M, Ghaderiyan GH, Shahin M, Navidifar T. First investigation of the presence of SPATE genes in Shigella species isolated from children with diarrhea infection in Ahvaz, southwest Iran. Infect Drug Resist. 2019;12: 795–804. doi:10.2147/IDR.S194740
