# Supplementary material for: Prevalence and changing antimicrobial resistance profiles of Shigella spp. isolated from diarrheal patients in Kolkata during 2011–2019
Source: PLoS Negl Trop Dis. 2024 Feb 20;18(2):e0011964. doi: 10.1371/journal.pntd.0011964 (PMC10906866; doi:10.1371/journal.pntd.0011964)
Supplement: S2 Table — (DOCX) [file pntd.0011964.s002.docx]

**S2 Table**

| ***Target*** | **Primer** | **Sequence (5’-3’)** | **Annealing**  **Temperature (°C)** | **Base pair** | **Reference** |
| --- | --- | --- | --- | --- | --- |
| *int1* | *int1_F* | CCTCCCGCACGATGATC | 57 | 280 | [1] |
|  | *int1_R* | TCCACGCATCGTCAGGC |  |  |  |
| *int2* | *int2_F* | CAGGGATATGCGACAAAAAGGT | 57 | 789 | [1] |
|  | *int2_R* | GTAGCAAACGAGTGACGAAATG |  |  |  |
| *int3* | *int3_F* | CGAATGCCCCAACAACTC | 57 | 922 | [1] |
|  | *int3_R* | ATCTGCCAAACCTGACTG |  |  |  |
| 3’ CS of class I integron | *qacE∆I* | ATCGCAATAGTTGGCGAAGT | 58 | 798 | [2] |
|  | *Sul1B* | GCAAGGCGGAAACCCGCC |  |  |  |
| Variable region of class 1 integron | *InF* | GGCATCCAAGCAGCAAGC | 60 | Variable | [1] |
|  | *InB* | AAGCAGACTTGACCTGAT |  |  |  |
| Variable region of atypical class 1 integron | *Int I CA F* | CGTAGAAGAACAGCAAGG | 52 | Variable | [2] |
|  | *ISI CA R* | AGTGAGAGCAGAGATAGC |  |  |  |
| Variable region of class 2 integron | *Int2 VA F* | CGGGATCCCGGACGGCATGCACGATTTGTA | 55 | Variable | [1] |
|  | *Int2 VA R* | GATGCCATCGCAAGTACGAG |  |  |  |
| *bla_OXA-1_* | *bla_OXA-1_*_F | GCAGCGCCAGTGCATCAAC | 50 | 198 | [3] |
|  | *bla_OXA-1_*_R | CCGCATCAAATGCCATAAGTG |  |  |  |
| *bla_TEM_* | *bla_TEM_*_F | CAT TTC CGT GTC GCC CTT ATT CC | 59 | 828 | [3] |
|  | *bla_TEM_*_R | GGC ACC TAT CTC AGC GAT CTG TCT A |  |  |  |
| *bla_CTX-M_* | *bla_CTX-M__*F | AATCACTGCGTCAGTTCAC | 59 | 701 | [3] |
|  | *bla_CTX-M__*R | TTTATCCCCCACAACCCAG |  |  |  |
| *Sul2* | *Sul2_F* | TTCGGCATTCTGAATCTCAC | 50 | 822 | [3] |
|  | *Sul2_R* | ATGATCTAACCCTCGGTCTC |  |  |  |
| *dfrIa* | *dfrIa_F* | GTGAAACTATCACTAATGG | 55 | 474 | [4] |
|  | *dfrIa_R* | TTAACCCTTTTGCCAGATTT |  |  |  |
| *cat* | *cat_F* | AAG TTG GCA GCA TTC ACC CG | 61 | 573 | [4] |
|  | *cat_R* | TCG TGG TAT TCA CTC CAG AGC G |  |  |  |
| *aadA* | *aadA_F* | AAC GAC CTT TTG GAA ACT TCG G | 60 | 352 | [5] |
|  | *aadA_R* | TTC GCT CAT CGC CAG CCC AG |  |  |  |
| *strA* | *strA_F* | CCA ATC GCA GAT AGA AGG CAA G | 65 | 580 | [3] |
|  | *strA_R* | ATC AAC TGG CAG GAG GAA CAG G |  |  |  |
| *tetA* | *tetA_F* | GGT CTT GCT CGT CTC GCT GG | 62 | 690 | [3] |
|  | *tetA_R* | AAC GCC ATC CAT CCC CGT G |  |  |  |
| *tetB* | *tetB_F* | CCTTATCATGCCAGTCTTGC | 50 | 774 | [3] |
|  | *tetB_R* | ACTGCCGTTTTTTCGCC |  |  |  |
| *qnrA* | *qnrA_F* | ATT TCT CAC GCC AGG ATT TG | 64 | 516 | [3] |
|  | *qnrA_R* | GAT CGG CAA AGG TTA GGT CA |  |  |  |
| *qnrB* | *qnrB_F* | GAT CGT GAA AGC CAG AAA GG | 64 | 476 | [3] |
|  | *qnrB_R* | ATG AGC AAC GAT GCC TGG TA |  |  |  |
| *qnrD* | *qnrD_F* | CGA GAT CAA TTT ACG GGG AAT A | 50 | 582 | [3] |
|  | *qnrD_R* | AAC AAG CTG AAG CGC CTG |  |  |  |
| *qnrS* | *qnrS_F* | GCA AGT TCA TTG AAC AGG GT | 64 | 428 | [3] |
|  | *qnrS_R* | TCT AAA CCG TCG AGT TGC GCG |  |  |  |
| *aac-(6’)-1b* | *aacIb_F* | TTG CGA TGC TCT ATG AGT GGC TA | 55 | 482 | [6] |
|  | *aacIb_R* | CTC GAA TGC CTG GCG TGT TT |  |  |  |
| *gyrA* | *gyrA_F* | TACACCGGTCAACATTGAGG | 64 | 648 | [2] |
|  | *gyrA_R* | TTAATGATTGCCGCCGTCGG |  |  |  |
| *gyrB* | *gyrB_F* | TGAAATGACCCGCCGTAAAGG | 60 | 310 | [2] |
|  | *gyrB_R* | GCTGTGATAACGCAGTTTGTCCGGG |  |  |  |
| *parC* | *parC_F* | GTCTGAACTGGGCCTGAATGC | 68 | 249 | [2] |
|  | *parC_R* | AGCAGCTCGGAATATTTCGACAA |  |  |  |
| *parE* | *parE_F* | ATGCGTGCGGCTAAAAAAGTG | 63 | 290 | [2] |
|  | *parE_R* | TCGTCGCTGTCAGGATCGATAC |  |  |  |

**Reference**

1. Zhu JY, Duan GC, Yang HY, Fan QT, Xi YL. Atypical Class 1 Integron Coexists with Class 1 and Class 2 Integrons in Multi-Drug Resistant Shigella flexneri Isolates from China. Curr Microbiol. 2011;62: 802–806. doi:10.1007/s00284-010-9790-3

2. Rajpara N, Nair M, Chowdhury G, Mukhopadhyay AK, Ramamurthy T, Niyogi SK, et al. Molecular analysis of multidrug resistance in clinical isolates of Shigella spp. from 2001-2010 in Kolkata, India: role of integrons, plasmids, and topoisomerase mutations. Infect Drug Resist. 2018;11: 87–102. doi:10.2147/IDR.S148726

3. Sarkar A, Pazhani GP, Dharanidharan R, Ghosh A, Ramamurthy T. Detection of integron-associated gene cassettes and other antimicrobial resistance genes in enterotoxigenic Bacteroides fragilis. Anaerobe. 2015;33: 18–24. doi:10.1016/j.anaerobe.2015.01.008

4. Marbou WJT, Jain P, Samajpati S, Halder G, Mukhopadhyay AK, Dutta S, et al. Profiling Virulence and Antimicrobial Resistance Markers of Enterovirulent Escherichia Coli from Fecal Isolates of Adult Patients with Enteric Infections in West Cameroon. PHRP. 2020;11: 216–230. doi:10.24171/j.phrp.2020.11.4.11

5. Maidhof H, Guerra B, Abbas S, Elsheikha HM, Whittam TS, Beutin L. A Multiresistant Clone of Shiga Toxin-Producing Escherichia coli O118:[H16] Is Spread in Cattle and Humans over Different European Countries. Appl Environ Microbiol. 2002;68: 5834–5842. doi:10.1128/AEM.68.12.5834-5842.2002

6. Park CH, Robicsek A, Jacoby GA, Sahm D, Hooper DC. Prevalence in the United States of aac(6′)-Ib-cr Encoding a Ciprofloxacin-Modifying Enzyme. Antimicrob Agents Chemother. 2006;50: 3953–3955. doi:10.1128/AAC.00915-06
