# Supplementary figures and images for: Prevalence and changing antimicrobial resistance profiles of Shigella spp. isolated from diarrheal patients in Kolkata during 2011–2019
Source: PLoS Negl Trop Dis. 2024 Feb 20;18(2):e0011964. doi: 10.1371/journal.pntd.0011964 (PMC10906866; doi:10.1371/journal.pntd.0011964)

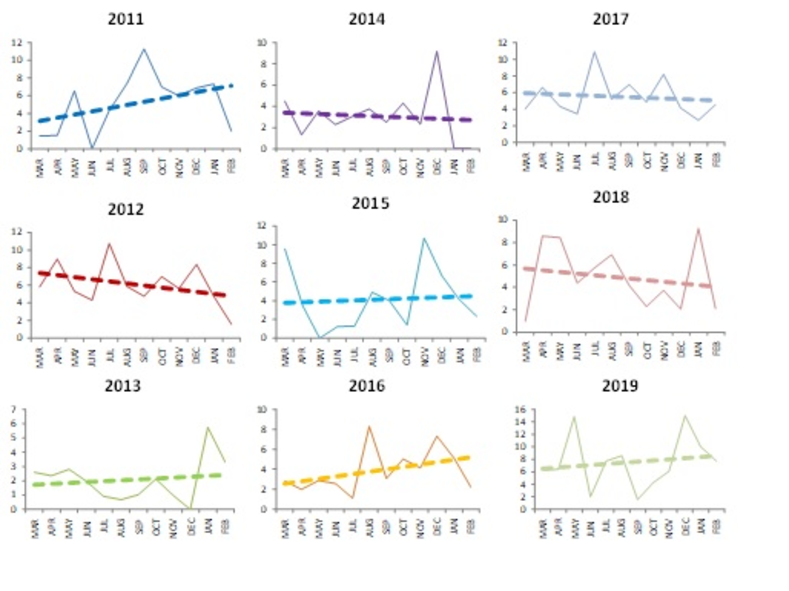

Supplement: S1 Fig — (TIF) [file pntd.0011964.s003.tif]

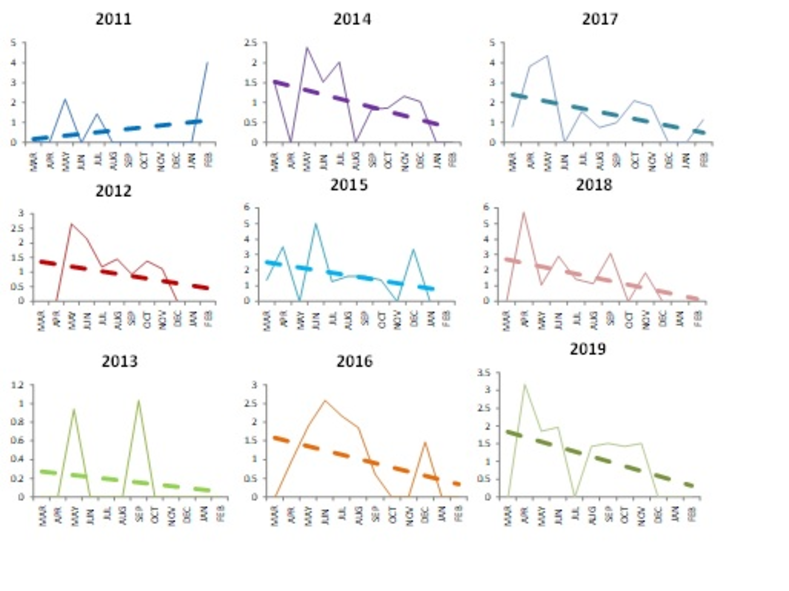

Supplement: S2 Fig — (TIF) [file pntd.0011964.s004.tif]

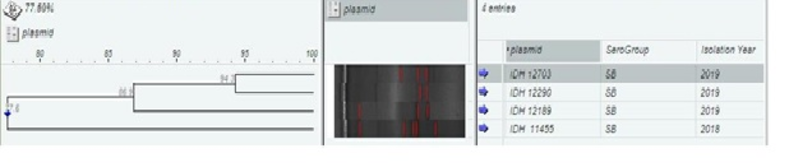

Supplement: S3 Fig — (TIF) [file pntd.0011964.s005.tif]

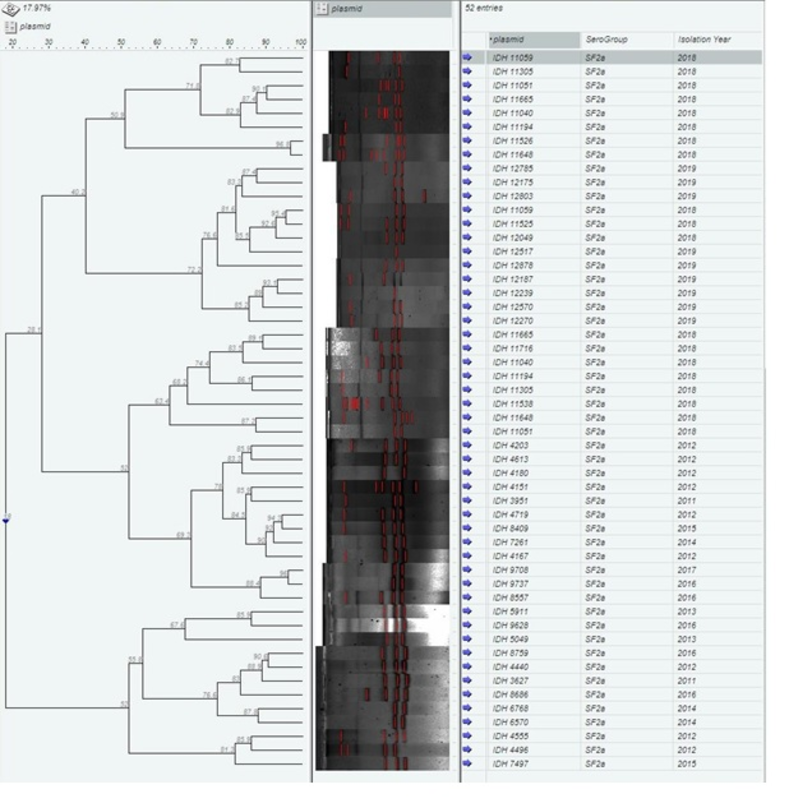

Supplement: S4 Fig — (TIF) [file pntd.0011964.s006.tif]

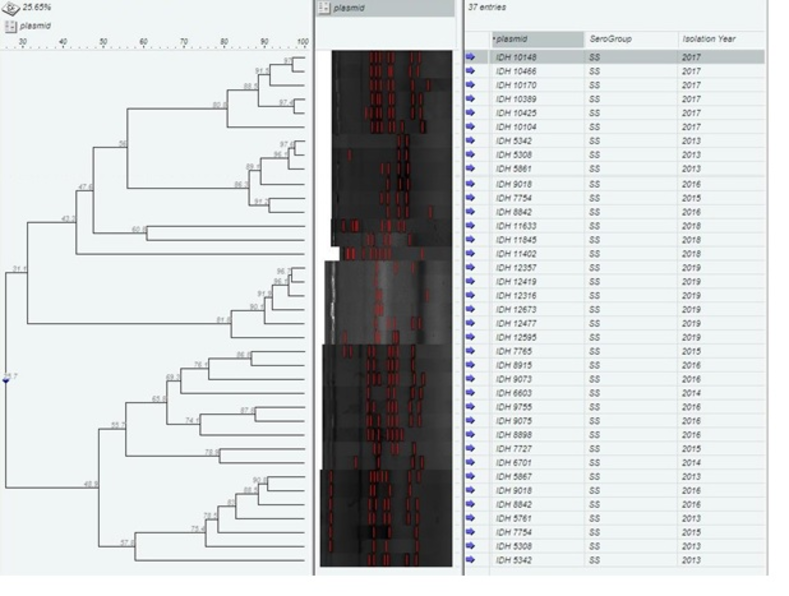

Supplement: S5 Fig — (TIF) [file pntd.0011964.s007.tif]
