# Supplementary material for: Prevalence and changing antimicrobial resistance profiles of Shigella spp. isolated from diarrheal patients in Kolkata during 2011–2019
Source: PLoS Negl Trop Dis. 2024 Feb 20;18(2):e0011964. doi: 10.1371/journal.pntd.0011964 (PMC10906866; doi:10.1371/journal.pntd.0011964)
Supplement: Attachment — Submitted filename: PNTD-D-23-01227_r.pdf [file pntd.0011964.s008.pdf]

PLOS Neglected Tropical Diseases

Prevalence and changing antimicrobial resistance profiles of Shigella spp. isolated from diarrheal patients in Kolkata during 2011-2019

--Manuscript Draft--

Manuscript Number:	PNTD-D-23-01227
Full Title:	Prevalence and changing antimicrobial resistance profiles of Shigella spp. isolated from diarrheal patients in Kolkata during 2011-2019
Short Title:	Prevalence and changing antimicrobial resistance profile of Shigella spp.
Article Type:	Research Article
Keywords:	Shigella; ipaH gene; Antimicrobial resistance; Surveillance
Abstract:	Background<br><br>The primary aim of this study was to investigate the occurrence, characteristics, and antimicrobial resistance patterns of various Shigella serotypes isolated from patients with acute diarrhea of the Infectious Diseases Hospital in Kolkata from 2011–2019.<br><br>Principal Findings<br><br>During the study period, Shigella isolates were tested for their serotypes, antibiotic resistance pattern and virulence gene profile. A total of 5.8% of Shigella spp. were isolated, among which S. flexneri (76.1%) was the highest, followed by S. sonnei (18.7%), S. boydii (3.4%), and S. dysenteriae (1.8%). Antimicrobial resistance against nalidixic acid was higher in almost all the Shigella isolates, while the resistance to β-lactamases, fluoroquinolones, tetracycline, and chloramphenicol diverged. The occurrence of multidrug resistance was found to be linked with various genesencoding drug-resistance, multiple mutations in the topoisomerase genes, and mobile genetic elements. All the isolates were positive for the invasion plasmid antigen H gene (ipaH). Dendrogram analysis of the plasmid and pulsed-field electrophoresis (PFGE) profiles revealed 70–80% clonal similarity among each Shigella serotype.<br><br>Conclusion<br><br>This comprehensive long-term surveillance report highlights the clonal diversity of clinical Shigella strains circulating in Kolkata, India, and shows alarming resistance trends towards recommended antibiotics. The elucidation of this study's outcome is helpful not only in identifying emerging antimicrobial resistance patterns of Shigella spp. but also in developing treatment guidelines appropriate for this region.
Additional Information:	
Question	Response
Government Employee<br><br>Are you or any of the contributing authors an employee of the United States government?<br><br>Manuscripts authored by one or more US Government employees are not copyrighted, but are licensed under a CC0 Public Domain Dedication, which allows unlimited distribution and reuse of the article for any lawful purpose. This is a	No - No authors are employees of the U.S. government.

Powered by Editorial Manager® and ProduXion Manager® from Aries Systems Corporation

legal requirement for US Government employees.<br><br>This will be typeset if the manuscript is accepted for publication.	
**Financial Disclosure**<br><br>Enter a financial disclosure statement that describes the sources of funding for the work included in this submission. Review the submission guidelines for detailed requirements. View published research articles from *PLOS Neglected Tropical Diseases* for specific examples.<br><br>This statement is required for submission and **will appear in the published article** if the submission is accepted. Please make sure it is accurate.<br><br>**Funded studies**<br>Enter a statement with the following details:<br>• Initials of the authors who received each award<br>• Grant numbers awarded to each author<br>• The full name of each funder<br>• URL of each funder website<br>• Did the sponsors or funders play any role in the study design, data collection and analysis, decision to publish, or preparation of the manuscript?<br><br>Did you receive funding for this work?	Yes
Please add funding details.<br>as follow-up to "**Financial Disclosure**<br><br>Enter a financial disclosure statement that describes the sources of funding for the work included in this submission. Review the submission guidelines for detailed requirements. View published research articles from *PLOS Neglected Tropical Diseases* for specific examples.<br><br>This statement is required for submission and **will appear in the published article** if	Japan and the Japan Agency for Medical Research and Development (AMED) [grant no. JP21wm0125004 (to SM)]

the submission is accepted. Please make sure it is accurate.<br><br>**Funded studies**<br>Enter a statement with the following details:<br>• Initials of the authors who received each award<br>• Grant numbers awarded to each author<br>• The full name of each funder<br>• URL of each funder website<br>• Did the sponsors or funders play any role in the study design, data collection and analysis, decision to publish, or preparation of the manuscript?<br><br>Did you receive funding for this work?"	
Please select the country of your main research funder (please select carefully as in some cases this is used in fee calculation).<br>as follow-up to "**Financial Disclosure**<br><br>Enter a financial disclosure statement that describes the sources of funding for the work included in this submission. Review the submission guidelines for detailed requirements. View published research articles from *PLOS Neglected Tropical Diseases* for specific examples.<br><br>This statement is required for submission and **will appear in the published article** if the submission is accepted. Please make sure it is accurate.<br><br>**Funded studies**<br>Enter a statement with the following details:<br>• Initials of the authors who received each award<br>• Grant numbers awarded to each author<br>• The full name of each funder<br>• URL of each funder website<br>• Did the sponsors or funders play any role in the study design, data collection and analysis, decision to publish, or preparation of the manuscript?	JAPAN - JP

Did you receive funding for this work?"	
**Competing Interests**<br><br>On behalf of all authors, disclose any competing interests that could be perceived to bias this work.<br><br>This statement will be typeset if the manuscript is accepted for publication.<br><br>Review the instructions link below and PLOS NTDs' competing interests policy to determine what information must be disclosed at submission.	No conflict of interest declared.
**Data Availability**<br><br>Provide a **Data Availability Statement** in the box below. This statement should detail where the data used in this submission can be accessed. This statement will be typeset if the manuscript is accepted for publication.<br><br>Before publication, authors are required to make all data underlying their findings fully available, without restriction. Review our PLOS Data Policy page for detailed information on this policy. Instructions for writing your Data Availability statement can be accessed via the Instructions link below.	The data that support the findings of this study are available from the corresponding author upon reasonable request.

Manuscript Click here to access/download;Manuscript;Shigella Manuscript_Sept 2023.docx

Prevalence and changing antimicrobial resistance profiles of *Shigella* spp. isolated from diarrheal patients in Kolkata during 2011-2019

Puja Bose[1], Goutam Chowdhury[1,3], Gourab Halder[1], Debjani Ghosh[1], Alok K Deb[2], Kei Kitahara[3,4], Shin-ichi Miyoshi[3,4], Masatomo Morita[5], Thandavarayan Ramamurthy[1], Shanta Dutta[1], Asish Kumar Mukhopadhyay[1]*

[1] Division of Bacteriology, ICMR-National Institute of Cholera and EntericDiseases,Kolkata, India

[2] Division of Epidemiology, ICMR-National Institute of Cholera and Enteric Diseases, Kolkata, India

[3] Collaborative Research Centre of Okayama University for Infectious Diseases at ICMR-NICED, Kolkata, India

[4] Graduate School of Medicine, Dentistry and Pharmaceutical Sciences, Okayama University, Okayama, Japan

[5] Department of Bacteriology I, National Institute of Infectious Diseases, Tokyo, Japan

*Corresponding author

E-mail: asish_mukhopadhyay@yahoo.com (AKM)

Abstract

Background: The primary aim of this study was to investigate the occurrence, characteristics, and antimicrobial resistance patterns of various *Shigella* serotypes isolated from patients with acute diarrhea of the Infectious Diseases Hospital in Kolkata from 2011–2019.

Principal Findings: During the study period, *Shigella* isolates were tested for their serotypes, antibiotic resistance pattern and virulence gene profile. A total of 5.8% of *Shigella* spp. were isolated, among which *S. flexneri* (76.1%) was the highest, followed by *S. sonnei* (18.7%), *S. boydii* (3.4%), and *S. dysenteriae* (1.8%). Antimicrobial resistance against nalidixic acid was higher in almost all the *Shigella* isolates, while the resistance to β-lactamases, fluoroquinolones, tetracycline, and chloramphenicol diverged. The occurrence of multidrug resistance was found to be linked with various genesencoding drug-resistance, multiple mutations in the topoisomerase genes, and mobile genetic elements. All the isolates were positive for the invasion plasmid antigen H gene (*ipaH*). Dendrogram analysis of the plasmid and pulsed-field electrophoresis (PFGE) profiles revealed 70–80% clonal similarity among each *Shigella* serotype.

Conclusion: This comprehensive long-term surveillance report highlights the clonal diversity of clinical *Shigella* strains circulating in Kolkata, India, and shows alarming resistance trends towards recommended antibiotics. The elucidation of this study's outcome is helpful not only in identifying emerging antimicrobial resistance patterns of *Shigella* spp. but also in developing treatment guidelines appropriate for this region.

Author Summary

Shigella spp. is one of the WHO-designated "antibiotic-resistant priority pathogens" that is the leading cause of bacterial diarrhea among children worldwide. The distribution of *Shigella* species varies depending on the geographic location, particularly in regions where environmental risk factors like substandard water quality, limited healthcare accessibility, and insufficient sanitation are widespread. The presence of serotype diversity and drug resistance combinations often leads to treatment failures. Therefore, it is crucial to comprehend the prevailing serogroups and antimicrobial resistance patterns of *Shigella* species in different geographical areas to implement effective treatment strategies. We have isolated 5.8% of *Shigella* spp. from 9276 diarrheal cases. Multidrug-resistant (MDR) *Shigella* globally requires a profound understanding of its genetic diversity, as there is significant regional variation in MDR strains. In our study, we observed a broad spectrum of resistance among the *Shigella* strains, with over 53.6% of the isolates demonstrating multidrug resistance. Representative isolates were evaluated for antimicrobial resistance encoding and virulence-associated genes as they enact significantly in disease development and interaction with the host immune system. This report marks the initial presentation of the outcomes from long-term surveillance and molecular characterization of antimicrobial resistances in clinical *Shigella* strains in Kolkata since 2011.The outcome of this investigations will guide in identifying emerging antimicrobial resistance trend, formulating suitable treatment guidelines tailored to the community's needs, and establishing baseline data for comparison with potential outbreak strains in the future.

Introduction

Shigella spp. is one of the listed "antibiotic-resistant priority pathogens" that pose a significant risk to global health (WHO, 2017) and are responsible for acute invasive intestinal infection leading to dysentery characterised by watery or bloody diarrhea [1,2]. *Shigella* spp. (*S. dysenteriae*, *S. flexneri*, *S. boydii*, and *S. sonnei*) is the etiological agent of shigellosis, and its species distribution varies spatio-temporally. The frequency of isolation of *S. flexneri* is more in developing and less developed countries, whereas *S. sonnei* has been reported as a major threat in developed regions [3]. Endemic and epidemic shigellosis are caused by many serotypes of *S. flexneri* and *S. dysenteriae* type 1 [4].

Globally, 165 million shigellosis cases have been reported every year in low- to middle-income countries, with more than 1 million deaths per year [5]. Access to clean water, good nutrition, sustained sanitation, and healthcare play a key role in reducing the disease burden [6,7]. *Shigella* has also been found to be the leading causative agent of childhood diarrhea, affecting 1.1 million children per year, predominantly infants to an age group of below 5 years old [8].

In addition to the characteristic of a low infective dose, a major role in the pathophysiology of the disease and its interaction with the host immune system was played by the virulence characteristics of *Shigella* [9]. Amidst numerous well-known virulence factors associated with *Shigella*spp,invasion plasmid antigen (Ipa) and the invasion-associated locus(Ial), facilitate intestinal cell penetration. The other factors, such as *Shigella* enterotoxin 1(ShET-1*)* and *Shigella* enterotoxin 2 (ShET-2) are encoded by the chromosomally-located *set* gene, *set1*, and the plasmid-encoded gene *sen*, respectively [10]. Plasmid-derived VirF and VirB proteins control invasion-related gene transcription. *Shigella* spp. harbor toxic factors like serine protease autotransporters of Enterobacteriaceae (SPATE) that include class 1 toxins,

Shigella IgA-like protease homologue (SigA), and secreted autotransporter toxin (Sat). The other is non-toxic SPATE class 2 toxins, which include the extracellular serine protease (*sepA*), whose association is mainly with intestinal inflammation, and colonisation [11]. Though several molecular methods are used for strain typing, pulsed-field electrophoresis (PFGE) is the most popular method to discriminate among clonal isolates [12].

Although *Shigella* seems to be a self-limiting disease, the WHO guidelines recommend antimicrobials in the treatment of the disease to control epithelial infiltration, duration of diarrhea, severity of symptoms, disease carrier stage, and death incidence [13]. Due to the emergence of multidrug-resistant (MDR) strains, shigellosis treatment has become more challenging. Consequently, MDR strains of *Shigella* are increasingly reported to be resistant to most of the recommended antimicrobials, like ciprofloxacin, third-generation cephalosporin, ceftriaxone, and macrolide azithromycin [8]. Recent data suggests that there are about 700,000 deaths worldwide every year as a result of antimicrobial resistance (AMR) [5].

AMR is also reported to be associated with a variety of biological, pharmacological, and social variables, like this organism's ability to acquire extra genetic material in the form of plasmids, pathogenicity islands, transposons, etc., as well as the indiscriminate antibiotic use [14–18]. Significant regional variation in MDR *Shigella* across the world makes it crucial to depend on information about its genetic diversity in determining epidemiological shifts as well as the development of appropriate treatment methods.

Different studies have been made across the world to track the epidemiology and antimicrobial susceptibility patterns of *Shigella* spp. In Kolkata, MDR *Shigella* spp. has shown to be increasing, especially towards the fluoroquinolone group of antimicrobials [19]. However, there has been a dearth of information related to prevalence and the AMR pattern in *Shigella* strains isolated from Kolkata and its surrounding areas since 2010. Therefore, the

current study was aimed to identify the epidemiological pattern of shigellosis as well as its AMR patterns in this region.

Materials and methods

1. Bacterial isolation and identification

From January 2011 through December 2019, 9276 enrolled patients with diarrhoea at Infectious Disease Hospital, Kolkata, were tested, and 535 (5.8%) were found to have *Shigella* spp. Stool samples were tested for common enteric pathogens using standard microbiological methods within 2 hours of collection [20]. Antisera from a commercial supplier (Denka Seiken Co. Ltd., Tokyo, Japan) were used in slide agglutination tests to verify the identification of *Shigella* spp. isolates based on biochemical methods. This research had previously been approved by the institution's ethics review board. Each isolate was preserved in a 15% glycerol stock that was frozen at -80˚C.

2. Antimicrobial susceptibility testing and determination of MIC

Shigella isolates were tested for their susceptibility to various antibiotics using the Kirby-Bauer disc-diffusion method on Mueller-Hinton agar, as recommended by the Clinical and Laboratory Standards Institute (https://www.nih.org.pk/wp-content/uploads/2021/02/CLSI-2020.pdf). Commercially available discs (Becton Dickinson, BBL): nalidixic acid (NA; 30µg), ciprofloxacin (CIP; 5µg), norfloxacin (NOR; 10µg), ofloxacin (OFX; 5µg), tetracycline (TET; 30µg), ampicillin (AM; 10µg), streptomycin (S; 10µg), trimethoprim-sulfamethoxazole (SXT; 1.25/23.75µg), ceftriaxone (CRO; 30µg) and chloramphenicol (CHL; 30µg) were used in this assay. Following the CLSI standards, we interpreted the data. For a subset of resistant

isolates, we used the agar dilution method to determine the CLSI-recommended minimum inhibitory concentrations (MICs) of nalidixic acid, ciprofloxacin, ampicillin, tetracycline, streptomycin, and chloramphenicol. In order to ensure consistent results, we used *Escherichia coli* ATCC 25922 as a reference strain.

3. Preparation of template DNA

Bacterial cells that had been cultured for 24 hours in a volume of 200µl of TE buffer were heated to 100 °C for 10 minutes and then stored on ice. After the thermal shock, a centrifuge was used to spin the resulting suspension at 10,000 rpm for 5 minutes. The template DNA for the PCR assay came from about 4 ul of the supernatant.

4. PCR amplification of virulence genes

The genes for virulence (*ipaH*, *ial*, *set* (sHET-1), *sen* (sHET-2), *virF*, *virB*, *sat*, *sigA*, *sep*, and *pic*) were identified by polymerase chain reaction (PCR) with the primers provided in **S1Table**.

5. Determination of integron and AMR gene markers by PCR

The three integrons (*intI*, *intII*, and *intIII*) were identified using PCR techniques. To further categorise the isolates into those with typical or atypical class 1 gene cassettes, we looked for the presence of the qacEΔ1 gene at the 3' end of the class 1 conserved segment and at the gene cassette within the variable region of class 1 and class 2 integrons. Direct sequencing of the PCR product was used to identify class 1 and class 2 resistance gene cassettes. The BLAST programme was used to compare the obtained sequences to those in the NCBI database, and the results were analysed accordingly.

In order to screen for the presence of specific resistance genes, PCR was performed on MDR *Shigella* isolates using previously published primers. (**Table S2**). Several

resistance genes like, β-lactamase genes (*bla*$_{OXA}$, *bla*$_{TEM}$); sulphonamide (*sul2*) and trimethoprim (*dfr1*) resistance genes; chloramphenicol (*catA*) resistance gene; streptomycin (*aadA*, *strA*); tetracycline (*tetA*, *tetB*) were mainly screened. In addition, the presence of plasmid-mediated quinolone resistance (PMQR) genes (*qnrA*, *qnrB*, *qnrD*, *qnrS*, and *aac-(6')-1b*) was investigated in some quinolone-resistant isolates. The role of topoisomerase mutations in quinolone resistance was investigated by using two or more representative isolates with differing profiles of quinolone resistance. The mutations were found by amplification and sequencing of the quinolone resistance determining regions (QRDRs) of topoisomerase (*gyrA*, *gyrB*, *parC*, and *parE*) and comparison to *Shigella* control strain DNA (Accession: NC_004741.1) from the database.

6. Plasmid profiling

Plasmids were extracted from representative isolates using a modified version of the Kado and Liu method [21]. Plasmid profiles were characterised by electrophoresis on an agarose gel with a dilution of 0.8%. After electrophoresis and ethidium bromide staining, the gels were photographed using a Bio-Rad gel documentation system. Using a DNA ladder as a standard, the migration patterns of the unknown plasmids were compared with those of the known plasmids, allowing their molecular weights to be calculated.

7. Analysis of similarity among isolates with the help of plasmid profiling

Dendrograms were constructed using the FPQuest software to show the degree to which the isolates were similar based on their plasmid profiles. The unweighted pair

group method (UPGMA analysis) was applied to the matrix similarity coefficients (1.5% position tolerance) to produce a dendrogram with the average linkage method.

8. Molecular typing of isolates by PFGE

Following the PulseNet standard protocol (https://www.cdc.gov/pulsenet/pdf/ecoli-shigella-salmonella-pfge-protocol-508c.pdf), DNA fingerprinting was carried out by PFGE of XbaI digested genomic DNA of the study strains using CHEF DRIII (Bio-Rad). The molecular DNA ladder consisted of XbaI-digested Salmonella enteric serovar Braenderup H9812. Gel images were recorded using a Gel-Documentation system (Bio-Rad), and PFGE profiles were analysed with BioNumerics software (Applied Maths, version 5.0). Similarity between bands was measured using the Dice coefficient, and correlation coefficients between clusters were calculated using the UPGMA method.

Results

1. Prevalence of *Shigella* spp.

During the study period, 535 (5.8%) *Shigella* spp. were isolated from 9,276 enrolled diarrheal cases. The numbers and percentage of different *Shigella* serotypes isolated were 407 (76.1%) *S. flexneri*, 100 (18.7%) *S. sonnei*, 18 (3.4%) *S. boydii*, and 10 (1.8%) *S. dysenteriae*. **Table 1** shows the yearly distribution of *Shigella*. The number of stool specimens collected each year started increasing in 2013 and remained same throughout the study period. The isolation rate of *Shigella* spp. remained relatively stable during the study period, but in 2013, the rate of isolation decreased drastically. In 2019, the *Shigella* infection rate reached its peak, while its lowest occurrence was

observed in 2013. In fact, approximately 4-fold increase in annual infection rate from 2.2% in 2013 to 9% in 2019 (**Fig 1**) was noted. There is a significant difference in the isolation rate ($p < 0.00001$). **Fig 2** illustrates the overall monthly isolation of all *Shigella* spp during 9 years, the trends in the *S. sonnei* isolation rate with relation to seasonality show a decreasing trend from 2012 onwards (**S1 Fig**), i.e., the isolation rates were high during the pre-monsoon season (March–May) and low during the winter season (December–February). *S. sonnei* showed a slightly reverse trend in the year 2011. No such trend was observed for *S. flexneri* (**S2 Fig**).

Table 1. Yearly distribution and infection of *Shigella*serotypes from stool sample of acute diarrheal patients at ID hospital, 2011-2019

**Year**	**2011**	**2012**	**2013**	**2014**	**2015**	**2016**	**2017**	**2018**	**2019**	**TOTA[illegible]**
Total no. of stool specimens	**643**	**967**	**1184**	**1135**	**1195**	**1266**	**1254**	**940**	**692**	**9276**
S.dysenteriae	**1(0.16)**	**2(0.2)**	**0(0)**	**1(0.09)**	**0(0)**	**4(0.32)**	**0(0)**	**1(0.11)**	**1(0.14)**	**10(0.1[illegible]**
S.flexneri	**31(4.82)**	**58(5.99)**	**21(1.77)**	**35(3.08)**	**46(3.84)**	**47(3.71)**	**71(5.66)**	**48(5.11)**	**50(7.23)**	**407(4.[illegible]**
S.boydii	**0(0)**	**5(0.52)**	**2(0.17)**	**5(0.44)**	**0(0)**	**1(0.08)**	**1(0.08)**	**1(0.11)**	**3(0.43)**	**18(0.1[illegible]**
S.sonnei	**4(0.62)**	**11(1.14)**	**3(0.25)**	**11(0.97)**	**19(1.59)**	**12(0.95)**	**19(1.52)**	**13(1.38)**	**8(1.16)**	**100(1.[illegible]**
Total no. isolated	**36(5.59)**	**76(7.86)**	**26(2.2)**	**52(4.58)**	**65(5.44)**	**64(5.06)**	**91(7.26)**	**63(6.70)**	**62(8.96)**	**535(5.[illegible]**

Fig1. Distribution of *Shigella* isolates in patients during 2011 and 2019 in Kolkata, India.*Shigella*infection rate of (%): the proportion of shigellosis cases in diarrheapatients.

Fig2. Seasonal distribution of total shigellosis cases.

The proportion of *Shigella* cases varied majorly by age groups, with the highest rate being 9% in 6–14 years of age group, followed by 7.2% in >60 years of age group. In

addition, the data represented predominance of *S. flexneri* infection (6.9%) in children aged between 6–14 years (**Fig 3**). *S. sonnei* infection dominates in the 6–14-year-old age group (1.9%), followed by <5 years of age (1.8%) (**Fig 3**). *S. flexneri* and *S. sonnei* isolation rates among the 0–5 year age group remained almost similar. Although there was no significant relation with respect to different serotype distribution among the other age groups (p = 0.873). However, the isolation rate of *S. sonnei* among the different age groups showed a significant variation (p = 0.0001).The isolation percentage among the 0–14 group was much higher than the other age groups. The S. *flexneri* isolation rate was significantly higher in age groups other than 0–5 years (p = 0.0001).In the case of *Shigella* spp., the isolation rate among males was slightly higher (56.8%) than that of female patients (43.2%).

Fig3. Demographic features of *Shigella* cases. Percentage (%): the proportion of *Shigella* cases by different groups of ages.

During the entire duration of the study, *S. flexneri* was the most predominant serotype, among which serotype 2a (54%) had the highest rate, followed by serotypes 3a, 4a, and 1b, with prevalence rates of 21%, 8%, and 5%, respectively **(Fig 4)**. Notably, 13 (4.2%) *S. flexneri* were designated as *S. flexneri* untypable (UT), as they only agglutinated with polyvalent type B antiserum; further serotyping of these isolates could not be completed with commercially available *Shigella* antisera.

Fig4. The serotypes of *S. flexneri* species from 2011 to 2019 in Kolkata, India.

2. Antimicrobial resistance in *Shigella* spp.

A significant portion of isolates exhibited resistance to nalidixic acid (91.8%), ciprofloxacin (83.4%), ofloxacin (81.9%), norfloxacin (79.8%), tetracycline (75.5%), co-trimoxazole (65.6%), and streptomycin (69.9%). For chloramphenicol and ampicillin, the *Shigella* isolates showed a medium range of resistance (46.3% and 54.4%, respectively). For the third generation cephalosporin, and ceftriaxone, the susceptibility rate of the isolates was about 94%. All the isolates remained susceptible to the carbapenem group of antibiotics **(Table 2)**. In *S. dysenteriae*, nalidixic acid and streptomycin resistance were high (80% and 70%, respectively). However, all *S. dysenteriae*isolates were susceptible to ceftriaxone.

Table 2. The percentages of resistance among different *Shigella*serotypes to various antimicrobial agents.

	**Number resistant to antimicrobials (%)**				
**Antimicrobial agents**	**Total *(n=535)***	***S. dysenteriae (n=10)***	***S. flexneri (n=407)***	***S. boydii (n=17)***	***S. sonnei (n=100)***
Nalidixic Acid	491(91.77)	8(80)	373(91.64)	11(64.70)	99(99)
Ciprofloxacin	446(83.36)	2(20)	342(84.02)	3(17.65)	99(99)
Norfloxacin	427(79.81)	1(10)	331(81.33)	3(17.65)	92(92)
Ofloxacin	438(81.87)	1(10)	340(83.54)	2(11.76)	95(95)
Tetracycline	404(75.51)	5(50)	329(80.84)	3(17.65)	67(67)
Ampicillin	291(54.39)	5(50)	262(64.37)	11(64.70)	13(13)
Ceftriaxone	32(5.98)	0(0)	25(6.14)	1(5.88)	6(6)
Co-trimoxazole	351(65.60)	5(50)	259(63.63)	10(58.82)	77(77)
Streptomycin	374(69.90)	7(70)	271(66.58)	16(94.12)	80(80)

Chloramphenicol	248(46.35)	3(30)	240(58.97)	1(5.88)	4(4)

Almost 80% of *S. flexneri* had multidrug resistance, whereas only 6.1% were resistant to ceftriaxone. *S. boydii* was susceptible to most of the tested drugs except streptomycin, nalidixic acid, ampicillin, and co-trimoxazole, with resistances of 94.1%, 64.7%, 64.7%, and 58.8%, respectively. On the other hand, *S. sonnei* isolates showed 70–90% resistance to all tested antimicrobials, except ampicillin, chloramphenicol, and ceftriaxone (13%, 4%, and 6%, respectively).

During this 9-year study period, the resistance rate of *S. flexneri* to ampicillin and ceftriaxone increased from 47.9% to 69.2% and 3.2% to 9.6%, respectively, whereas that of *S. sonnei* increased from 0% to 17.1% and 0% to 6.9%, respectively. There was a notable increasein resistance to ciprofloxacin (p=0.000), ofloxacin (p=0.058), ampicillin (p=0.0088) and ceftriaxone (p=0.0106). There is a decreasing trend of resistance to co-trimoxazole. While there was no significant change in the *S. sonnei* AMR trend. *S. flexneri* isolates showed significant changes in the resistance for ampicillin, co-trimoxazole, and ceftriaxone (p= 0.0001, 0.0004, and 0.0105, respectively).

The MIC range for different serotypes is shown in **Table 3**. Several types of resistance profiles were observed, and the common resistance profiles have been depicted in **Table 4.**The majority of the *Shigella* isolates demonstrated resistance to multiple categories of antibiotics. The most common resistance profile of the isolates confered resistance to almost five groups of antibiotics, like aminoglycosides, tetracycline, antifolate, fluoroquinolones, and β-lactam.

Table 3. Minimum inhibitory concentrations MIC of antimicrobial agents for *Shigella* spp.

Antimicrobial agent	Breakpoint (µg/ml)	Range	*S. dysenteriae*		*S. flexneri*		*S. boydii*		*S. sonnei*	
			MIC_{50} (µg/ml)	MIC_{90} (µg/ml)	MIC_{50} (µg/ml)	MIC_{90} (µg/ml)	MIC_{50} (µg/ml)	MIC_{90} (µg/ml)	MIC_{50} (µg/ml)	MIC_{90} (µg/ml)
Ampicillin	≥32	32-512	>512	>512	>256-<512	>512-<1024	>128-<256	>512	>32-<64	>32-<64
Tetracycline	≥16	16-256	>64-<128	>64-<128	>64-<128	>64-<128	>16-<32	>32-<64	>64-<128	>64-<128
Ciprofloxacin	≥4	4-64	>4-<8	>4-<8	>4-<8	>16-<32	<4	<4	>4-<8	>8-<16
Nalidixic Acid	≥32	32-1024	>128-<256	>128-<256	>1024	>1024	>32-<64	>256-<512	>512-<1024	>512-<1024
Streptomycin	-	64-1024	>64-<128	>64-<128	>512-<1024	>1024	>64-<128	>64-<128	>256-<512	>1024
Chloramphenicol	≥32	32-512	<32	<32	>32-<64	>64-<128	<32	>32-<64	>16-<32	>32-<64

Table 4. Most common Resistance profile of *Shigella* isolates of ID hospital during 2011-2019

Sl No	Resistance profile	Total no. of Isolates	2011	2012	2013	2014	2015	2016	2017	2018	2019
**I**	**NA/CIP/NOR/OFX/TET/AM/SXT/S/CHL**	**100**	**12**	**22**	**3**	**7**	**11**	**12**	**12**	**10**	**11**
**II**	**NA/CIP/NOR/OFX/TET/SXT/S**	**70**	**5**	**11**	**2**	**7**	**13**	**9**	**14**	**5**	**4**

**III**	**NA/CIP/NOR/OFX/TET/AM /S/CHL**	**57**	**0**	**4**	**0**	**6**	**5**	**3**	**21**	**11**	**7**
**IV**	**NA/CIP/NOR/OFX/TET/SX T/S/CHL**	**38**	**8**	**10**	**3**	**2**	**1**	**1**	**7**	**2**	**4**
**V**	**NA/CIP/NOR/OFX/TET/AM /SXT/S**	**22**	**5**	**0**	**0**	**1**	**7**	**1**	**2**	**2**	**4**
**VI**	**NA/CIP/NOR/OFX**	**17**	**1**	**2**	**1**	**1**	**3**	**2**	**3**	**2**	**2**
**VII**	**NA/CIP/NOR/OFX/S**	**14**	**0**	**1**	**1**	**0**	**0**	**3**	**5**	**1**	**3**

3. Detection of virulence genes

About 51% of the isolates harboured *ipaH,* which was encoded in an invasive plasmid. *virF* and *virB* were found in 50.9% and 40.8% of the isolates, respectively. The chromosomally encoded *set* was present in 39.5% of the tested isolates, whereas the plasmid-encoded *sen* was found in 48.4% of the isolates. The secreted autotransporter toxin-encoded *sat* gene was found in 52.2% of the isolates, and other serine protease autotransporter toxin encoding genes like *sigA*, *pic*, and *sepA* were present in 63%, 35.7%, and 53.5% of the isolates, respectively.

4. Characterisation of integrons and AMR genes

A total of 28.6%, 26.12%, and 40.13% of the *Shigella* isolates harboured *intlI, intlII,* and both *intlI/intlII* genes, respectively. No class *intIII* was noted in any of the *Shigella* isolates. The prevalence of *intI, intII* and both *intI/intII* genes in different serotypes of *Shigella* spp. is shown in **Fig 5.**

Fig5. Distribution of different intrgron in different serotypes of *Shigella.*

The presence of atypical class 1integrons and typical class 1 integrons was detected in 17.8% and 5% of the *Shigella*isolates, respectively. While 17.4% of the isolates were found to carry both class 1 typical and class 1 atypical integrons. Interestingly, different types of class 1 integrons co-existed with class 2 integrons, with 16.6% of the isolates carrying the combination of atypical class 1/class 2integrons, while only 7% of the isolates had typical class 1/class 2 integrons (**Fig 6**).

Fig 6. Distribution of different class of integron among tested isolates.

The strains with typical integrons showed two different sizes of amplicons (500 bp and >2.5 kb). On sequencing, the 500 bp of the class 1 integron did not show any of the antimicrobial resistance gene cassette. Atypical class 1 integrons had 2 kb and 2.4 kb amplicons sizes, and upon sequencing, the 2kb had 100% nucleotide identity with GenBank accession no. GQ214137 of *S. flexneri* with the AMR gene cassettes *dfrA1-aadA*, and *bla*$_{OXA}$-*aadA*gene cassettes with full sequence similarity with GenBank accession no. KX817769 was carried by 2.4 kb variable region of the atypical class 1 integron. Two different sizes of class 2 gene cassettes were found among the tested isolates. The 1.4 kb cassette was observed mainly in *S.sonne*i, whereas the 2.2kb region was found in other *Shigella* spp. The former had a sequence similarity *dfrA1-sat* (GenBank accession no. KX817766),and the later shared the *dfrA1-sat-aadA* sequence similarity (GenBank accession no. KX817767).

The presence of AMR genes was screened among isolates that were resistant to one or more antimicrobials. Among these, 69.4% were positive for *bla*$_{OXA-1}$, whereas only 12.3% were positive for *bla*$_{TEM-9}$. *tetA* and *tetB* genes were respectively detected in 13.9% and 77.9% of the isolates that confer the resistance to tetracycline. *qnrB* (4.6%), *qnrS* (3.8%),and *aac(6')1b* (0.8%) were found to be responsible for

fluoroquinolone resistance. Mostly, quinolone resistance was associated with *gyrA* (S83L, D87N, H211Y) and/or *parC*(S80I) mutations (**Table 5**). No mutation was seen in the *gyrB* and *parE*. Other AMR-encoding genes like *strA* and *aadA1A* for streptomycin and *dfrA1* and *sul2* for co-trimoxazole were detected in 52.4%, 55.8%, 84%, and 61.9%, respectively, whereas the prevalence of the *catA*, responsible for chloramphenicol resistance was found 46.1% of the isolates.

Table 5. Mutations in quinolone resistance determining regions (QRDR) of representative quinolone resistant *Shigella* isolates

Strain	Strain ID	Quinolone resistance	MIC CIP(µg/ml)	*GyrA*	*ParC*
S. flexneri 2a	IDH10994	NA, CIP, NOR, OFX	4-8	S83->L D87->N H211->Y	S80->I
S. sonnei	IDH 8898	NA, CIP, NOR, OFX	-16	S83->L D87->N	S80->I
S. flexneri 2a	IDH 10932	NA, CIP, NOR, OFX	16-32	S83->L D87->N H211->Y	S80->I
S. dysenteriae	IDH 9602	NA, CIP, NOR, OFX	2-4	-	-
S. sonnei	IDH 6701	NA, CIP, NOR, OFX	0.5-1	S83->L D87->G	S80->I

5. Plasmid Profiling

The dendrogram-based genetic similarity analysis of representative *Shigella* spp. was analysed**.** There was a similarity of 77.6% among the *S. boydii* group (**S3 Fig**),

whereas the similarities were less among *S. flexneri* and *S. sonnei* (18% and 25.6%, respectively) (**S4 and S5 Figs**), indicating the diversity of plasmid patterns.

6. Molecular typing by PFGE

The *Xba*I DNA fragments obtained in the PFGE were analysed with the corresponding species: *S. dysenteriae* (**Fig 7**), *S. flexneri* (**Fig 8**), *S. boydii* (**Fig 9**) and *S. sonnei* (**Fig 10**). PFGE analysis of *S. dysenteriae* isolates revealed two clusters with approximately 70% similarity within them (**Fig 7**). When *S. flexneri*2a isolates of different years and AMR profiles were compared, there were two clusters present with 85% similarity (**Fig 8**). The IDH 12673 isolate was identified as belonging to a different clone with only 74% similarity to other *S. flexneri* 2a. Among the 9 *S. boydii* isolates, 6 strains formed a distinct cluster with 76% similarity to another cluster of 3 isolates (**Fig 9**). *S. sonnei* had two clusters with 85% similarity. Among 32 representative isolates, 18 belonged to the first cluster, whereas another 14 strains belonged to the second cluster and shared 90–100% similarity among the isolates **(Fig 10)**. Only IDH 7727 shows a unique banding pattern with 83% similarity to the other isolates.

Fig7. *XbaI*-PFGE profiles of with dendrogram showing percentage similarity of *S. dysenteriae* isolates.

Fig 8. *XbaI*-PFGE profiles of with dendrogram showing percentage similarity of *S. flexneri* isolates.

Fig 9. *XbaI*-PFGE profiles of with dendrogram showing percentage similarity of *S. boydii* isolates.

Fig 10. *XbaI*-PFGE profiles of with dendrogram showing percentage similarity of *S. sonnei* isolates.

Discussion

The frequency of *Shigella* isolation, the species distribution, and the antimicrobial resistance pattern were examined in this study. The total isolation rate of was 5.8%, with *S. flexneri* (76.1%) being the most dominant one, followed by *S. sonnei* (18.7%), *S. boydii* (3.4%), and *S. dysenteriae* (1.8%). A similar trend has been reported from Bangladesh and China [22,23]. Our finding indicates that the overall isolation rate of *Shigella* spp. from Kolkata remains 6-7% and the prevalence of its species remained the same as reported before from the same region [19,24]. Two investigations conducted in Kolkata between 2001 and 2007 [25,26] and one conducted in North Karnataka over a period of 12 years [27] found that the frequency of *S. dysenteriae* was 2.1% greater than that of *S. boydii*, which slightly differs from our findings. Numerous investigations around the world [28–33] revealed that the most common serotype was*S. flexneri* 2a, which is also observed in this study. We found that the children belonged to the age group 6–14 years had the largest proportion of being infected with *S. flexneri* or *S. sonnei*. A study conducted in Asia and Africa indicated that children aged below 5 years are mostly infected with *Shigella* spp. [34,35]. One plausible explanation for the lack of cases in children younger than 5 years old in current study might be due to the fact that children with mild to moderate diarrhea would have received treatment at home or in other hospitals in the city. Like in other reports [36,37], the ratio of male to female *Shigella*-infected cases is similar.

Shigella pathogenesis is the result of a combination of several different virulence factors. Almost all the species of *Shigella* had plasmid-borne *inv* and also the chromosomally encoded virulence factors.

In 2017, the WHO highlighted the urgent need for new antibiotics on a list of priority pathogens, and *Shigella* was among them. More than 60% of the isolates in our investigation were resistant to fluoroquinolone, aminoglycoside, and folate inhibitory groups, which

matched with other global reports [38–42]. However, *S. sonnei* was more sensitive to ampicillin and chloramphenicol than *S. flexneri*. Ceftriaxone and azithromycin are currently recommended by the WHO for the treatment of fluoroquinolone-resistant *Shigella* infections. Our finding indicates that *Shigella* was highly susceptible to cephalosporin, suggesting that this drug could be a better choice for the empirical treatment.

According to a CDC's report, approximately 77,000 drug-resistant *Shigella* infections occur annually, making this pathogen categorized as a serious threat [43]. Determining the role of genetic elements such as integrons, plasmids, and chromosomally carried topoisomerase mutations in the development of highly multi-drug-resistant phenotypes is therefore crucial. Certain *Shigella* isolates have developed resistance to antibiotics like trimethoprim and spectinomycin because of integrons [44,45]. Previous studies have indicated that a majority of the isolates examined had an atypical class 1 integron, but that a few had the typical class 1 integron [46]. In class two integrons, *dfrA1-sat* and *dfrA1-sat-aadA* are the most common gene cassettes identified in *S. sonnei* and *S. flexneri*, respectively. Class 2 integrons seem to be the distinctive feature of these two *Shigella* spp.

The mobile genetic elements, including integrons and plasmids are playing a significant role in the spread of AMR among bacteria. These bacteria frequently have the bla_{TEM}, which imparts resistance to penicillin and other β-lactam antibiotics [47,48]. Nevertheless, the OXA-type β-lactam, with significant hydrolytic activity against oxacillin and cloxacillin, frequently transmits resistance to ampicillin and cephalothin. Similar to the other studies, our findings show that 69.4% of the isolates tested positive for $bla_{\text{OXA-1}}$ and 12.3% for bla_{TEM} [49,50]. In accordance with earlier results [49], *tet(B)* was more frequent than *tet(A)*. Streptomycin resistance was largely attributed to *strA*, but *aadA1* also appears to play a strong role. Quinolone resistance is typically caused by mutations in QRDR region, efflux pump activity, and PMQR genes [51]. In this study, several mutations in the QRDR have

been identified, irrespective of MIC. Mutations in *gyrA* (S83, D87, H211) and *parC* (S80) have been frequently reported. An association of broader range of resistance to different quinolone was also observed with mutations in both topoisomerases [52].

Our study has identified the presence of the PMQR genes *qnrB*, *qnrS*, and *aac(6')1b* that are responsible for fluoroquinolone resistance. Empirical treatments rely heavily on antibiotic resistance patterns since it can also be used as a typing system and AMR determinants spreading indicator as well [53]. Due to the absence of diversity in the susceptibility patterns, we were unable to use the AMR pattern as an epidemiological marker.

Molecular epidemiological investigations, comparing plasmid and PFGE profiles, are valuable tools for determining the probable relatedness among clinical isolates of a specific bacterial species [54–56]. Our study ascertained that the clonal relatedness among *S. dysenteriae* isolates was 70%, which is almost similar to previous reports from Kolkata [19,46,57]. In *S. flexneri*, 85% similarity was noted, which is also similar to other findings [19,57–60]. As shown in a previous study from Kolkata, representative isolates of *S. boydii* had 76% similarity [46]. The clonal relatedness of *S. sonnei* from different countries reproduces a parallel observation like our study outcome [22,49,50,60–62]. As evidenced from this study, multiple clones were identified in different serotypes, confirming the existence of diverse origins among *Shigella* spp.

Acknowledgements

This research was supported in part by the Indian Council of Medical Research (ICMR), Govt. Of India; the National Institute of Infectious Diseases (NIID), Japan and the Japan Agency for Medical Research and Development (AMED) [grant no. JP21wm0125004 (to SM)]. GC acknowledges the ‘Scientist C’ position received from the Okayama University, Okayama, Japan. TR is the Indian National Science Academy (INSA) Senior Scientist. We

are thankful to Mr. Mukul Roy and Mr. Biswajit Sharma for technical assistance in the PFGE experiment.

References

1. Nüesch-Inderbinen M, Heini N, Zurfluh K, Althaus D, Hächler H, Stephan R. Shigella Antimicrobial Drug Resistance Mechanisms, 2004–2014. Emerg Infect Dis. 2016;22: 1083–1085. doi:10.3201/eid2206.152088

2. Tacconelli E, Carrara E, Savoldi A, Harbarth S, Mendelson M, Monnet DL, et al. Discovery, research, and development of new antibiotics: the WHO priority list of antibiotic-resistant bacteria and tuberculosis. Lancet Infect Dis. 2018;18: 318–327. doi:10.1016/S1473-3099(17)30753-3

3. Ghosh S, Pazhani GP, Niyogi SK, Nataro JP, Ramamurthy T. Genetic characterization of Shigella spp. isolated from diarrhoeal and asymptomatic children. J Med Microbiol. 2014;63: 903–910. doi:10.1099/jmm.0.070912-0

4. Venkatesan MM, Ranallo RT. Live-attenuated Shigella vaccines. Expert Rev Vaccines. 2006;5: 669–686. doi:10.1586/14760584.5.5.669

5. Hussen S, Mulatu G, Yohannes Kassa Z. Prevalence of Shigella species and its drug resistance pattern in Ethiopia: a systematic review and meta-analysis. Ann Clin Microbiol Antimicrob. 2019;18: 22. doi:10.1186/s12941-019-0321-1

6. Schroeder GN, Hilbi H. Molecular Pathogenesis of Shigella spp.: Controlling Host Cell Signaling, Invasion, and Death by Type III Secretion. Clin Microbiol Rev. 2008;21: 134–156. doi:10.1128/CMR.00032-07

7. Taneja N, Mewara A. Shigellosis: Epidemiology in India. Indian J Med Res. 2016;143: 565–576. doi:10.4103/0971-5916.187104

8. Williams PCM, Berkley JA. Guidelines for the treatment of severe acute malnutrition: a systematic review of the evidence for antimicrobial therapy. Paediatr Int Child Health. 2018;38: S32–S49. doi:10.1080/20469047.2017.1409453

9. Fan W, Qian H, Shang W, Ying C, Zhang X, Cheng S, et al. Low distribution of genes encoding virulence factors in Shigella flexneri serotypes 1b clinical isolates from eastern Chinese populations. Gut Pathogens. 2017;9: 76. doi:10.1186/s13099-017-0222-9

10. Casabonne C, González A, Aquili V, Balagué C. Prevalence and Virulence Genes of Shigella spp. Isolated from Patients with Diarrhea in Rosario, Argentina. Jpn J Infect Dis. 2016;69: 477–481. doi:10.7883/yoken.JJID.2015.459

11. Parham NJ, Pollard SJ, Desvaux M, Scott-Tucker A, Liu C, Fivian A, et al. Distribution of the Serine Protease Autotransporters of the Enterobacteriaceae among Extraintestinal Clinical Isolates of Escherichia coli. J Clin Microbiol. 2005;43: 4076–4082. doi:10.1128/JCM.43.8.4076-4082.2005

12. European Centre for Disease Prevention and Control. Annual epidemiological report 2013 :reporting on 2011 surveillance data and 2012 epidemic intelligence data. LU: Publications Office; 2013. Available: https://data.europa.eu/doi/10.2900/13174

13. Ashkenazi S. Shigella infections in children: new insights. Semin Pediatr Infect Dis. 2004;15: 246–252. doi:10.1053/j.spid.2004.07.005

14. Rowe-Magnus DA, Guerout A-M, Mazel D. Bacterial resistance evolution by recruitment of super-integron gene cassettes. Mol Microbiol. 2002;43: 1657–1669. doi:10.1046/j.1365-2958.2002.02861.x

15. Al-Nimri S, Miller WA, Byrne BA, Guibert G, Chen L. A unified approach to molecular epidemiology investigations: tools and patterns in California as a case study for endemic shigellosis. BMC Infectious Diseases. 2009;9: 184. doi:10.1186/1471-2334-9-184

16. Farthing M, Salam MA, Lindberg G, Dite P, Khalif I, Salazar-Lindo E, et al. Acute diarrhea in adults and children: a global perspective. J Clin Gastroenterol. 2013;47: 12–20. doi:10.1097/MCG.0b013e31826df662

17. Porter CK, Thura N, Ranallo RT, Riddle MS. The Shigella human challenge model. Epidemiol Infect. 2013;141: 223–232. doi:10.1017/S0950268812001677

18. Das A, Natarajan M, Mandal J. The Emergence of Quinolone Resistant Shigella sonnei, Pondicherry, India. PLoS One. 2016;11: e0160290. doi:10.1371/journal.pone.0160290

19. Ghosh S, Pazhani GP, Chowdhury G, Guin S, Dutta S, Rajendran K, et al. Genetic characteristics and changing antimicrobial resistance among Shigella spp. isolated from hospitalized diarrhoeal patients in Kolkata, India. J Med Microbiol. 2011;60: 1460–1466. doi:10.1099/jmm.0.032920-0

20. Nair GB, Ramamurthy T, Bhattacharya MK, Krishnan T, Ganguly S, Saha DR, et al. Emerging trends in the etiology of enteric pathogens as evidenced from an active surveillance of hospitalized diarrhoeal patients in Kolkata, India. Gut Pathog. 2010;2: 4. doi:10.1186/1757-4749-2-4

21. Kado CI, Liu ST. Rapid procedure for detection and isolation of large and small plasmids. J Bacteriol. 1981;145: 1365–1373. doi:10.1128/jb.145.3.1365-1373.1981

22. Ud-Din AIMS, Wahid SUH, Latif HA, Shahnaij M, Akter M, Azmi IJ, et al. Changing trends in the prevalence of Shigella species: emergence of multi-drug resistant Shigella sonnei biotype g in Bangladesh. PLoS One. 2013;8: e82601. doi:10.1371/journal.pone.0082601

23. Yang H, Chen G, Zhu Y, Liu Y, Cheng J, Hu L, et al. Surveillance of Antimicrobial Susceptibility Patterns among Shigella Species Isolated in China during the 7-Year Period of 2005-2011. Ann Lab Med. 2013;33: 111–115. doi:10.3343/alm.2013.33.2.111

24. Dutta S, Ghosh A, Ghosh K, Dutta D, Bhattacharya SK, Nair GB, et al. Newly Emerged Multiple-Antibiotic-Resistant Shigella dysenteriae Type 1 Strains in and around Kolkata, India, Are Clonal. Journal of Clinical Microbiology. 2003;41: 5833. doi:10.1128/JCM.41.12.5833-5834.2003

25. Pazhani GP, Ramamurthy T, Mitra U, Bhattacharya SK, Niyogi SK. Species diversity and antimicrobial resistance of Shigella spp. isolated between 2001 and 2004 from hospitalized children with diarrhoea in Kolkata (Calcutta), India. Epidemiol Infect. 2005;133: 1089–1095. doi:10.1017/S0950268805004498

26. Nandy S, Mitra U, Rajendran K, Dutta P, Dutta S. Subtype prevalence, plasmid profiles and growing fluoroquinolone resistance in Shigella from Kolkata, India (2001–2007): a hospital-based study. Tropical Medicine & International Health. 2010;15: 1499–1507. doi:10.1111/j.1365-3156.2010.02656.x

27. Jain PA, Kulkarni RD, Dutta S, Ganavali AS, Kalabhavi AS, Shetty PC, et al. Prevalence and antimicrobial profile of Shigella isolates in a tertiary care hospital of North Karnataka: A 12-year study. Indian J Med Microbiol. 2020;38: 101–108. doi:10.4103/ijmm.IJMM_20_107

28. Mamatha B, Pusapati BR, Rituparna C. Changing patterns of antimicrobial susceptibility of Shigella serotypes isolated from children with acute diarrhea in Manipal, South India, a 5 year study. Southeast Asian J Trop Med Public Health. 2007;38: 863–866.

29. Bhattacharya D, Bhattacharya H, Thamizhmani R, Sayi DS, Reesu R, Anwesh M, et al. Shigellosis in Bay of Bengal Islands, India: clinical and seasonal patterns, surveillance of antibiotic

susceptibility patterns, and molecular characterization of multidrug-resistant Shigella strains isolated during a 6-year period from 2006 to 2011. Eur J Clin Microbiol Infect Dis. 2014;33: 157–170. doi:10.1007/s10096-013-1937-2

30. Khaghani S, Shamsizadeh A, Nikfar R, Hesami A. Shigella flexneri: a three-year antimicrobial resistance monitoring of isolates in a Children Hospital, Ahvaz, Iran. Iran J Microbiol. 2014;6: 225–229.

31. Cui X, Wang J, Yang C, Liang B, Ma Q, Yi S, et al. Prevalence and antimicrobial resistance of Shigella flexneri serotype 2 variant in China. Front Microbiol. 2015;6: 435. doi:10.3389/fmicb.2015.00435

32. Liu H, Zhu B, Qiu S, Xia Y, Liang B, Yang C, et al. Dominant serotype distribution and antimicrobial resistance profile of Shigella spp. in Xinjiang, China. PLoS One. 2018;13: e0195259. doi:10.1371/journal.pone.0195259

33. Das A, Mandal J. Extensive inter-strain diversity among clinical isolates of Shigella flexneri with reference to its serotype, virulence traits and plasmid incompatibility types, a study from south India over a 6-year period. Gut Pathogens. 2019;11: 33. doi:10.1186/s13099-019-0314-9

34. Kotloff KL, Nataro JP, Blackwelder WC, Nasrin D, Farag TH, Panchalingam S, et al. Burden and aetiology of diarrhoeal disease in infants and young children in developing countries (the Global Enteric Multicenter Study, GEMS): a prospective, case-control study. The Lancet. 2013;382: 209–222. doi:10.1016/S0140-6736(13)60844-2

35. Zhao Z, Chen Q, Wang Y, Chu M, Hu Q, Hannah MN, et al. Relative transmissibility of shigellosis among different age groups: A modeling study in Hubei Province, China. PLoS Negl Trop Dis. 2021;15: e0009501. doi:10.1371/journal.pntd.0009501

36. Karacan C, Tavil B, Topal Y, Zorlu P, Tayman C. Evaluation of shigellosis in a Turkish children's hospital. Pediatr Int. 2007;49: 589–592. doi:10.1111/j.1442-200X.2007.02425.x

37. Mook P, Gardiner D, Kanagarajah S, Kerac M, Hughes G, Field N, et al. Use of gender distribution in routine surveillance data to detect potential transmission of gastrointestinal infections among men who have sex with men in England. Epidemiol Infect. 2018;146: 1468–1477. doi:10.1017/S0950268818001681

38. Peirano G, Souza F dos S, Rodrigues D dos P. Frequency of serovars and antimicrobial resistance in Shigella spp. from Brazil. Mem Inst Oswaldo Cruz. 2006;101: 245–250. doi:10.1590/S0074-02762006000300003

39. Heffernan H, Woodhouse R, Hewison C, Sherwood J. Antimicrobial resistance among Shigella in New Zealand. N Z Med J. 2018;131: 56–62.

40. Pakbin B, Amani Z, Allahyari S, Mousavi S, Mahmoudi R, Brück WM, et al. Genetic diversity and antibiotic resistance of Shigella spp. isolates from food products. Food Sci Nutr. 2021;9: 6362–6371. doi:10.1002/fsn3.2603

41. Phiri AFND, Abia ALK, Amoako DG, Mkakosya R, Sundsfjord A, Essack SY, et al. Burden, Antibiotic Resistance, and Clonality of Shigella spp. Implicated in Community-Acquired Acute Diarrhoea in Lilongwe, Malawi. Trop Med Infect Dis. 2021;6: 63. doi:10.3390/tropicalmed6020063

42. Gaufin T, Blumenthal J, Ramirez-Sanchez C, Mehta S, Pride DT, Fierer J, et al. Antimicrobial-Resistant Shigella spp. in San Diego, California, USA, 2017–2020. Emerg Infect Dis. 2022;28: 1110–1116. doi:10.3201/eid2806.220131

43. Centers for Disease Control and Prevention (U.S.). Antibiotic resistance threats in the United States, 2019. Centers for Disease Control and Prevention (U.S.); 2019 Nov. doi:10.15620/cdc:82532

44. Gassama Sow A, Diallo MH, Gatet M, Denis F, Aïdara-Kane A, Ploy M-C. Description of an unusual class 2 integron in Shigella sonnei isolates in Senegal (sub-Saharan Africa). Journal of Antimicrobial Chemotherapy. 2008;62: 843–844. doi:10.1093/jac/dkn264

45. Chang C-Y, Lu P-L, Lin C-C, Lee T-M, Tsai M-Y, Chang L-L. Integron types, gene cassettes, antimicrobial resistance genes and plasmids of Shigella sonnei isolates from outbreaks and sporadic cases in Taiwan. J Med Microbiol. 2011;60: 197–204. doi:10.1099/jmm.0.022517-0

46. Rajpara N, Nair M, Chowdhury G, Mukhopadhyay AK, Ramamurthy T, Niyogi SK, et al. Molecular analysis of multidrug resistance in clinical isolates of Shigella spp. from 2001-2010 in Kolkata, India: role of integrons, plasmids, and topoisomerase mutations. Infect Drug Resist. 2018;11: 87–102. doi:10.2147/IDR.S148726

47. Bradford PA. Extended-Spectrum β-Lactamases in the 21st Century: Characterization, Epidemiology, and Detection of This Important Resistance Threat. Clin Microbiol Rev. 2001;14: 933–951. doi:10.1128/CMR.14.4.933-951.2001

48. Barlow M, Hall BG. Experimental prediction of the natural evolution of antibiotic resistance. Genetics. 2003;163: 1237–1241. doi:10.1093/genetics/163.4.1237

49. Pazhani GP, Niyogi SK, Singh AK, Sen B, Taneja N, Kundu M, et al. Molecular characterization of multidrug-resistant Shigella species isolated from epidemic and endemic cases of shigellosis in India. J Med Microbiol. 2008;57: 856–863. doi:10.1099/jmm.0.2008/000521-0

50. Wang Y, Ma Q, Hao R, Zhang Q, Yao S, Han J, et al. Antimicrobial resistance and genetic characterization of Shigella spp. in Shanxi Province, China, during 2006–2016. BMC Microbiology. 2019;19: 116. doi:10.1186/s12866-019-1495-6

51. Atta-ur-Rahman Prof, Iqbal Choudhary M, editors. Therapeutic Limitations due to Antibiotic Drug Resistance: Road to Alternate Therapies. Frontiers in Anti-Infective Drug Discovery. BENTHAM SCIENCE PUBLISHERS; 2014. pp. 72–141. doi:10.2174/9781608059126114030004

52. Ding J, Ma Y, Gong Z, Chen Y. [A study on the mechanism of the resistance of Shigellae to fluoroquinolones]. Zhonghua Nei Ke Za Zhi. 1999;38: 550–553.

53. Farshad S, Sheikhi R, Japoni A, Basiri E, Alborzi A. Characterization of Shigella Strains in Iran by Plasmid Profile Analysis and PCR Amplification of ipa Genes. J Clin Microbiol. 2006;44: 2879–2883. doi:10.1128/JCM.00310-06

54. Ribot EM, Fair MA, Gautom R, Cameron DN, Hunter SB, Swaminathan B, et al. Standardization of pulsed-field gel electrophoresis protocols for the subtyping of Escherichia coli O157:H7, Salmonella, and Shigella for PulseNet. Foodborne Pathog Dis. 2006;3: 59–67. doi:10.1089/fpd.2006.3.59

55. Li S, Wang J, Wei X, Liu Y, You L, Luo X, et al. Molecular Characterization of Shigella sonnei: An Increasingly Prevalent Etiologic Agent of Shigellosis in Guizhou Province, Southwest of China. PLoS One. 2016;11: e0156020. doi:10.1371/journal.pone.0156020

56. Zhu Z, Wang W, Cao M, Zhu Q, Ma T, Zhang Y, et al. Virulence factors and molecular characteristics of Shigella flexneri isolated from calves with diarrhea. BMC Microbiol. 2021;21: 214. doi:10.1186/s12866-021-02277-0

57. Dutta S, Jain P, Nandy S, Matsushita S, Yoshida S-I. Molecular characterization of serologically atypical provisional serovars of Shigella isolates from Kolkata, India. J Med Microbiol. 2014;63: 1696–1703. doi:10.1099/jmm.0.081307-0

58. Viñas MR, Tuduri E, Galar A, Yih K, Pichel M, Stelling J, et al. Laboratory-based prospective surveillance for community outbreaks of Shigella spp. in Argentina. PLoS Negl Trop Dis. 2013;7: e2521. doi:10.1371/journal.pntd.0002521

59. Azmi IJ, Khajanchi BK, Akter F, Hasan TN, Shahnaij M, Akter M, et al. Fluoroquinolone Resistance Mechanisms of Shigella flexneri Isolated in Bangladesh. PLoS One. 2014;9: e102533. doi:10.1371/journal.pone.0102533

60. Ezernitchi AV, Sirotkin E, Danino D, Agmon V, Valinsky L, Rokney A. Azithromycin non-susceptible Shigella circulating in Israel, 2014–2016. PLOS ONE. 2019;14: e0221458. doi:10.1371/journal.pone.0221458

61. Nandy S, Dutta S, Ghosh S, Ganai A, Rajahamsan J, Theodore RBJ, et al. Foodborne-associated Shigella sonnei, India, 2009 and 2010. Emerg Infect Dis. 2011;17: 2072–2074. doi:10.3201/eid1711.110403

62. Cheng T, Shi X, Yong W, Wang J, Xie G, Ding J. Molecular typing of Shigella sonnei isolates circulating in Nanjing, China, 2007-2011. J Infect Dev Ctries. 2014;8: 1525–1532. doi:10.3855/jidc.4933

63. Wang S-J, Chen JH. A rapid and specific PCR method for the detection of Shigella spp. in spiked samples. Journal of Food and Drug Analysis. 2020;20. doi:10.38212/2224-6614.2081

64. Moosavian M, Ghaderiyan GH, Shahin M, Navidifar T. First investigation of the presence of SPATE genes in Shigella species isolated from children with diarrhea infection in Ahvaz, southwest Iran. Infect Drug Resist. 2019;12: 795–804. doi:10.2147/IDR.S194740

65. Zhu JY, Duan GC, Yang HY, Fan QT, Xi YL. Atypical Class 1 Integron Coexists with Class 1 and Class 2 Integrons in Multi-Drug Resistant Shigella flexneri Isolates from China. Curr Microbiol. 2011;62: 802–806. doi:10.1007/s00284-010-9790-3

66. Sarkar A, Pazhani GP, Dharanidharan R, Ghosh A, Ramamurthy T. Detection of integron-associated gene cassettes and other antimicrobial resistance genes in enterotoxigenic Bacteroides fragilis. Anaerobe. 2015;33: 18–24. doi:10.1016/j.anaerobe.2015.01.008

67. Marbou WJT, Jain P, Samajpati S, Halder G, Mukhopadhyay AK, Dutta S, et al. Profiling Virulence and Antimicrobial Resistance Markers of Enterovirulent Escherichia Coli from Fecal Isolates of Adult Patients with Enteric Infections in West Cameroon. PHRP. 2020;11: 216–230. doi:10.24171/j.phrp.2020.11.4.11

68. Maidhof H, Guerra B, Abbas S, Elsheikha HM, Whittam TS, Beutin L. A Multiresistant Clone of Shiga Toxin-Producing Escherichia coli O118:[H16] Is Spread in Cattle and Humans over Different European Countries. Appl Environ Microbiol. 2002;68: 5834–5842. doi:10.1128/AEM.68.12.5834-5842.2002

69. Park CH, Robicsek A, Jacoby GA, Sahm D, Hooper DC. Prevalence in the United States of aac(6')-Ib-cr Encoding a Ciprofloxacin-Modifying Enzyme. Antimicrob Agents Chemother. 2006;50: 3953–3955. doi:10.1128/AAC.00915-06

Supporting information Captions

S1 Table. Primer list for virulence gene.

S2 Table. Primer list for antimicrobial resistance gene.

S1 Fig. Trends for *S. sonnei* isolation rate with relation to seasonality.

S2 Fig. The trends for *S.flexneri* isolation rate with relation to seasonality.

S3 Fig. Similarity among the *Shigella boydii*isolates on the basis of their plasmid profiles.

S4 Fig. Similarity among the *Shigella flexneri 2a* isolates on the basis of their plasmid profiles.

S5 Fig. Similarity among the *Shigella sonnei* isolates on the basis of their plasmid profiles.

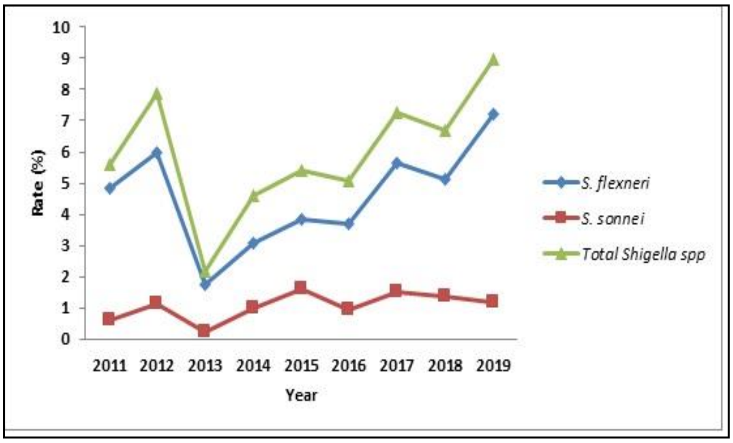

Fig1.Distribution of *Shigella* isolates in patients during 2011 and 2019 in Kolkata, India.

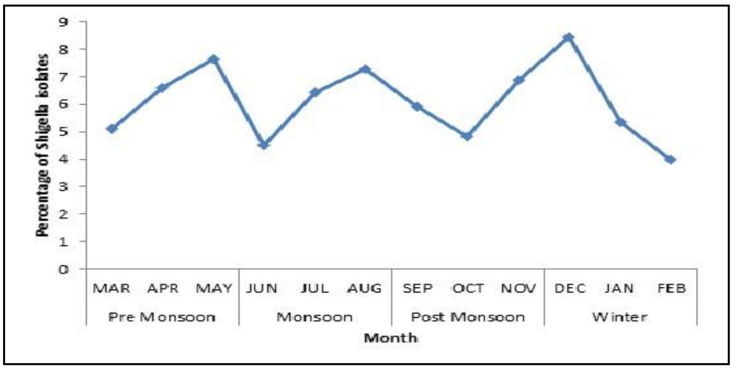

Fig 2. Seasonal distribution of total shigellosis cases.

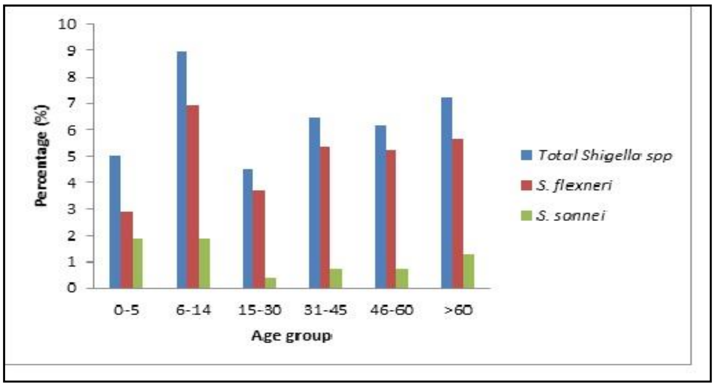

Fig 3. Demographic features of *Shigella* cases.

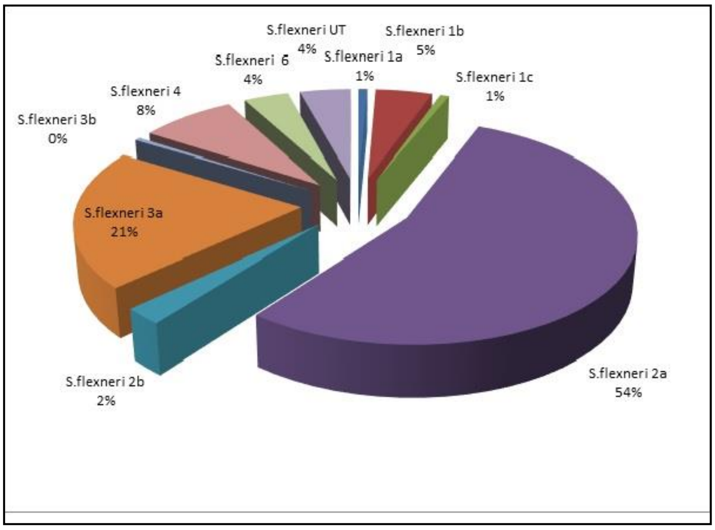

Fig 4. The serotypes of *S. flexneri* species from 2011 to 2019 in Kolkata, India.

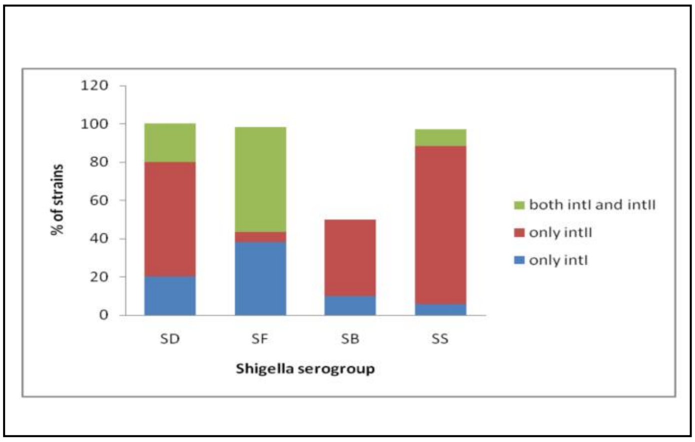

Fig5. Distribution of different intrgron in different serotypes of *Shigella.*

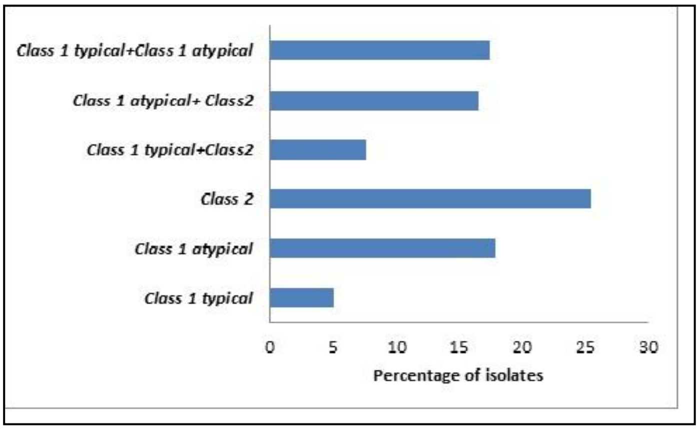

Fig 6.Distribution of different class of integron among tested isolates.

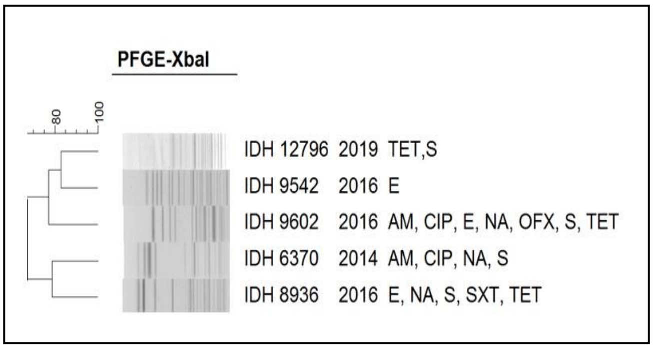

Fig 7. ***XbaI*-PFGE profiles of with dendrogram showing percentage similarity of *S. dysenteriae* isolates.**

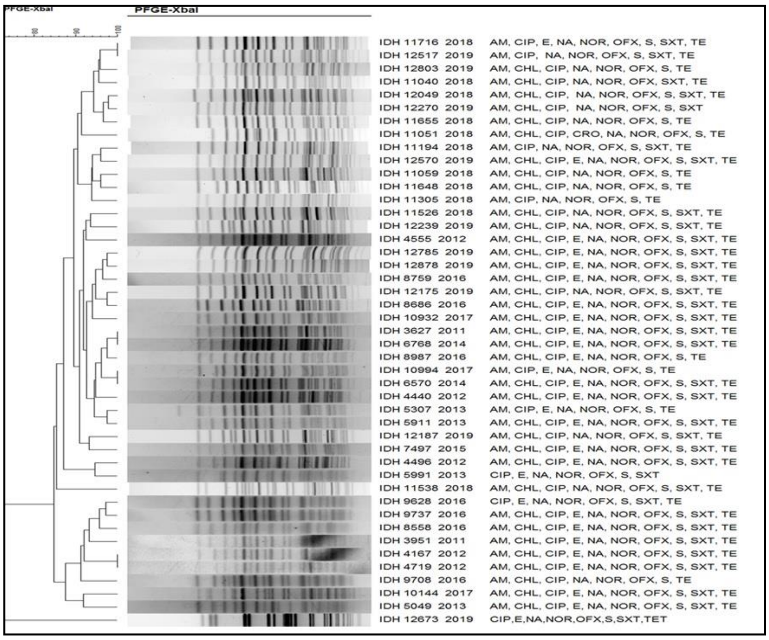

Fig 8. ***XbaI*-PFGE profiles of with dendrogram showing percentage similarity of *S. flexneri* isolates.**

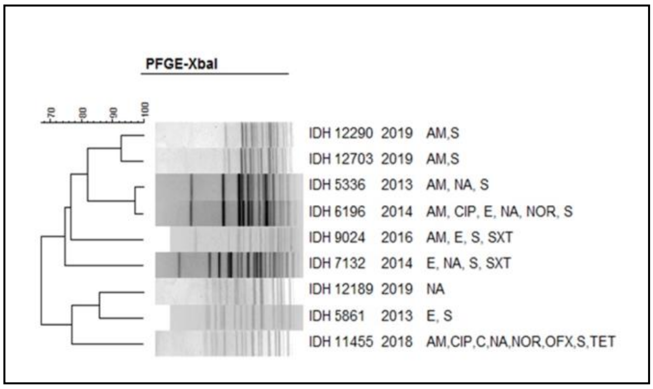

Fig 9. ***XbaI*-PFGE profiles of with dendrogram showing percentage similarity of *S. boydii* isolates.**

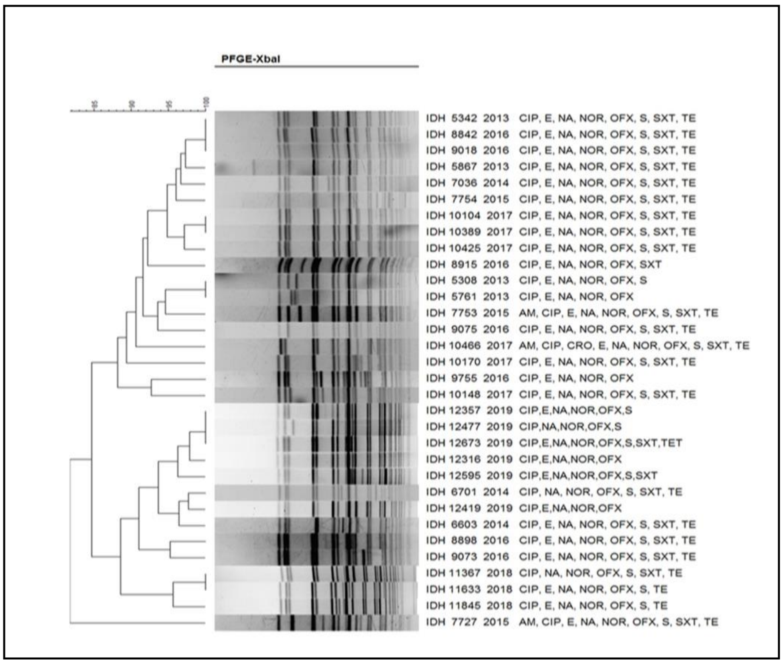

Fig 10. *XbaI*-PFGE profiles of with dendrogram showing percentage similarity of *S. sonnei*isolates.

Figure 1

Click here to access/download;Figure;Fig 1.tif

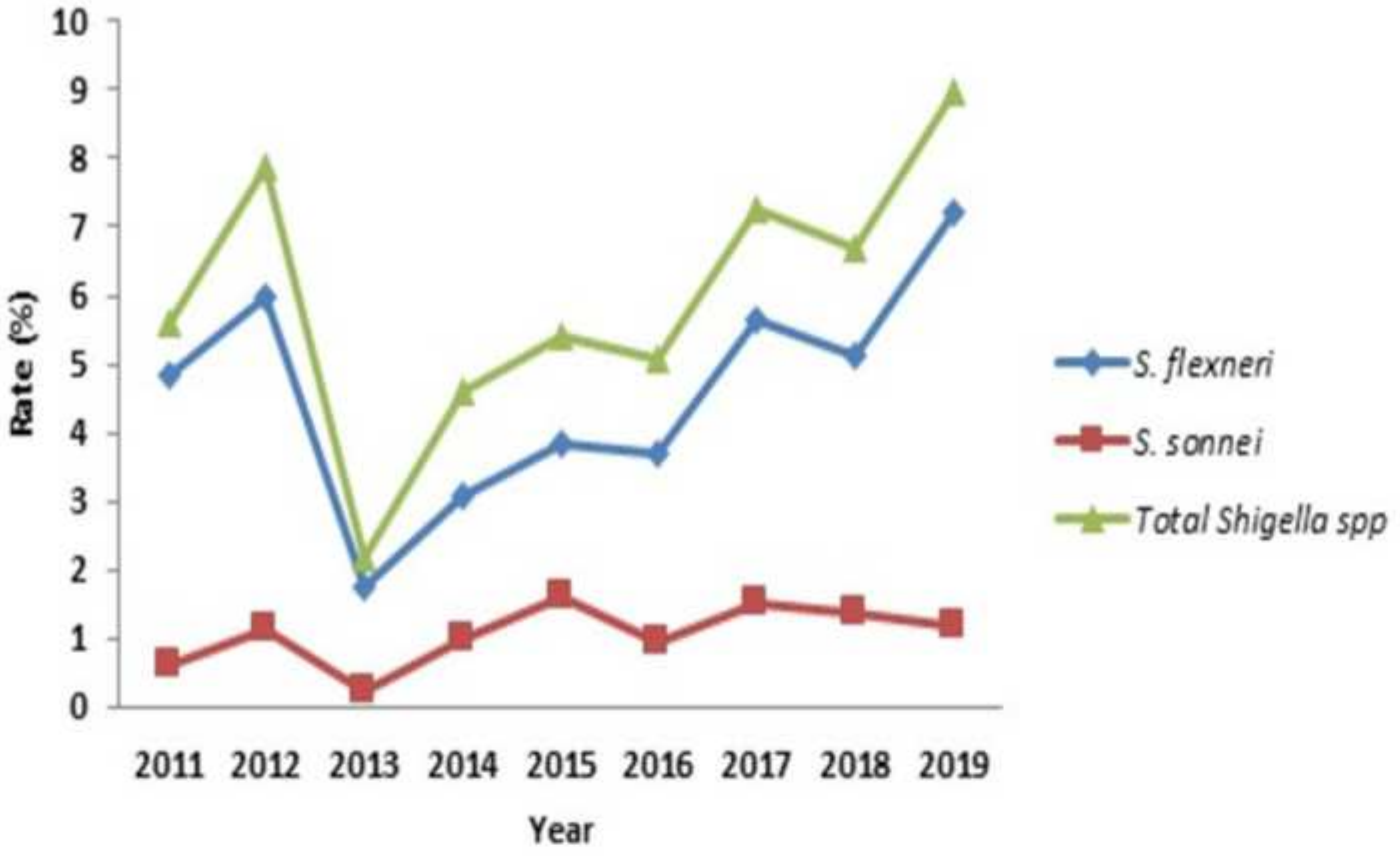

Figure 2

Click here to access/download;Figure;Fig 2.tif

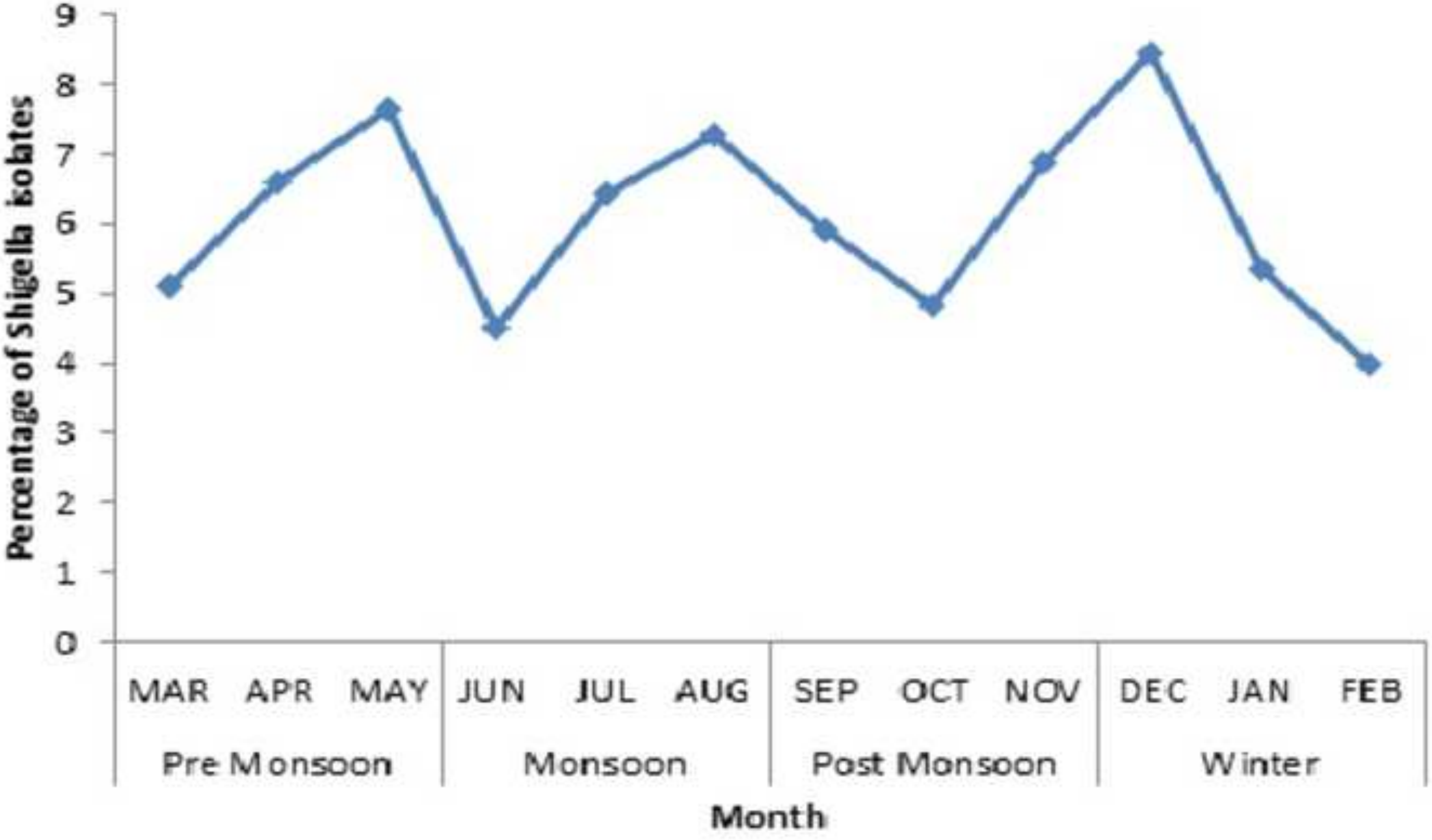

Figure 3 Click here to access/download;Figure;Fig 3.tif

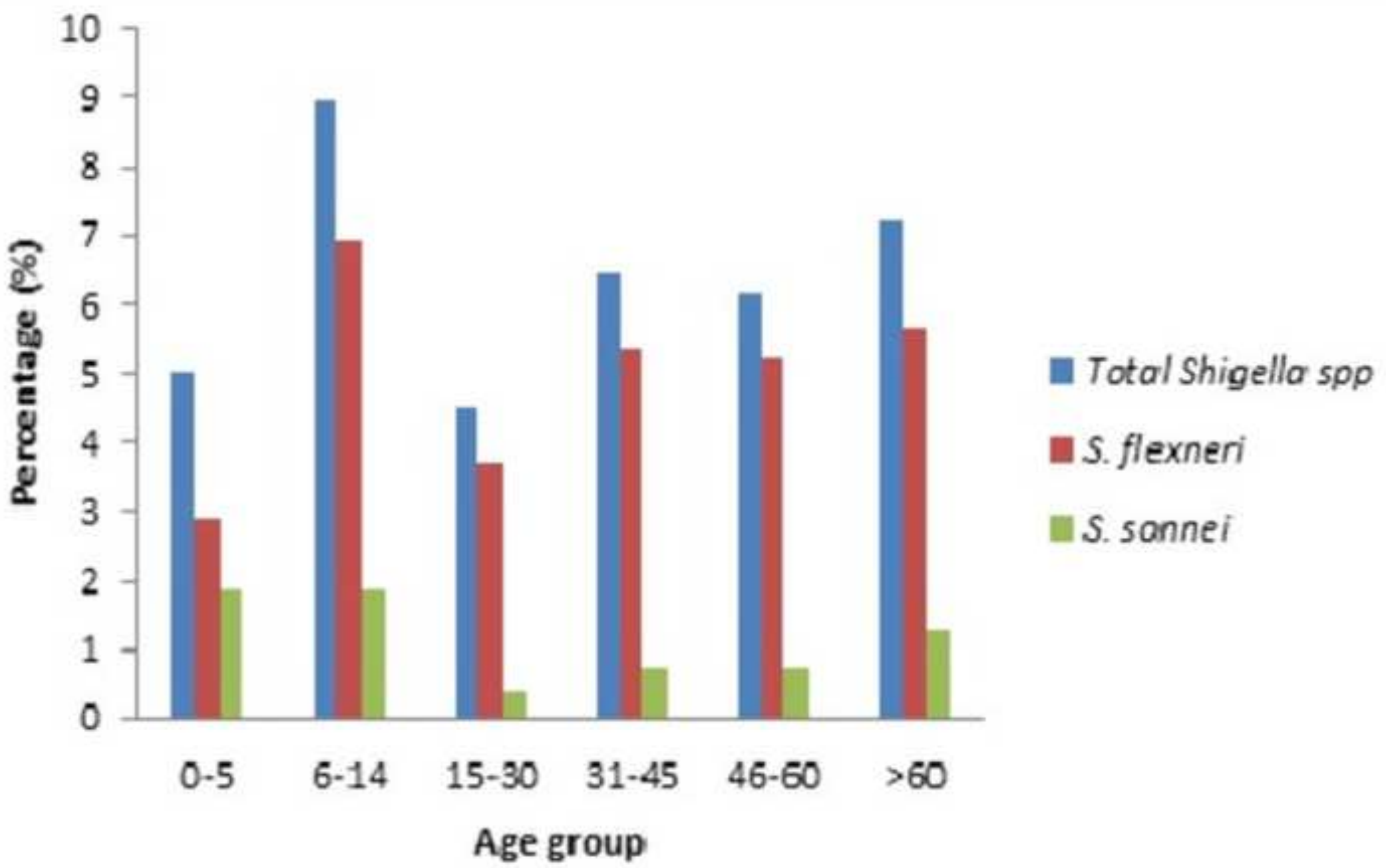

Figure 4

Click here to access/download;Figure;Fig 4.tif

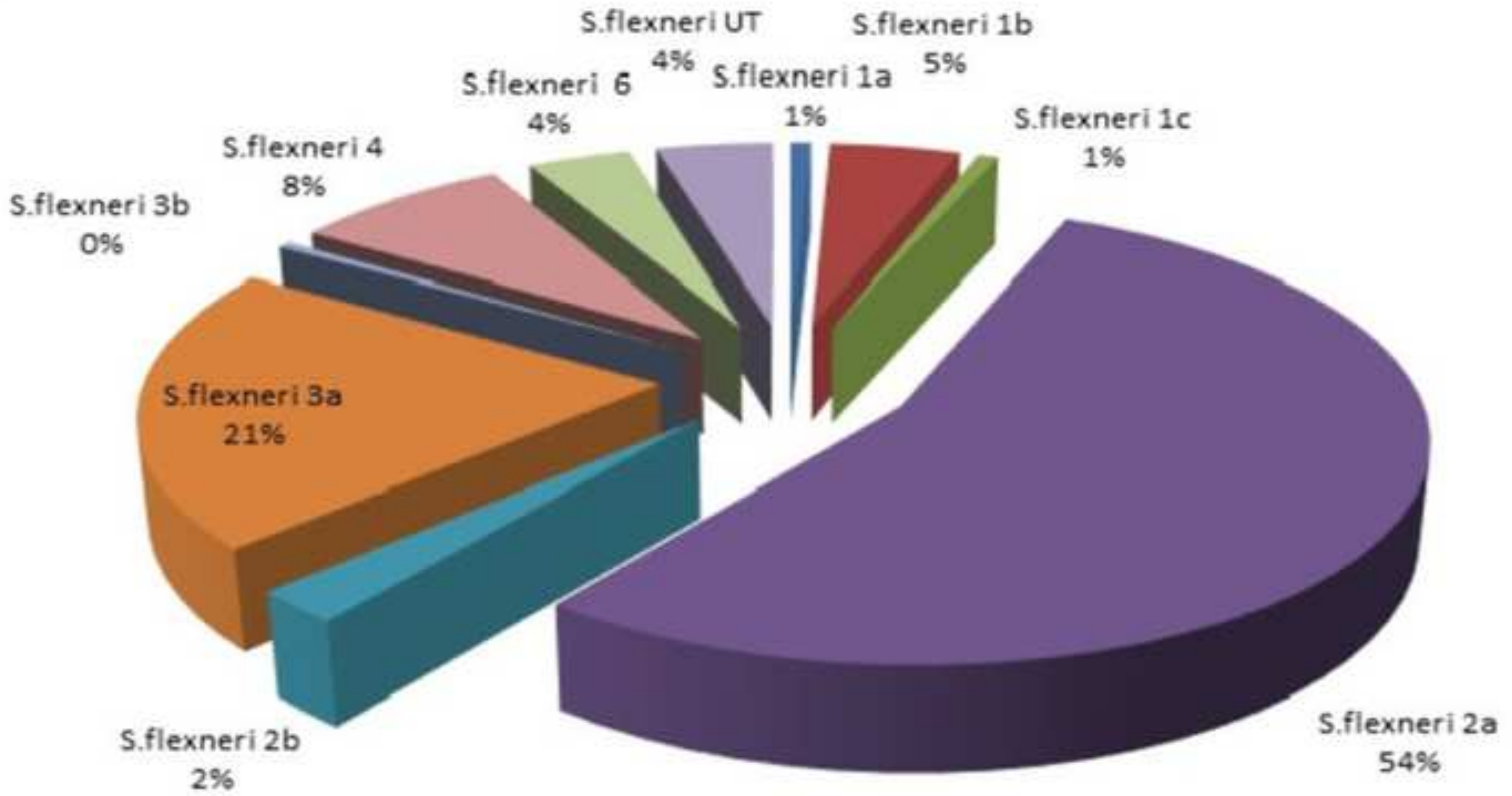

Figure 5

Click here to access/download;Figure;Fig 5.tif

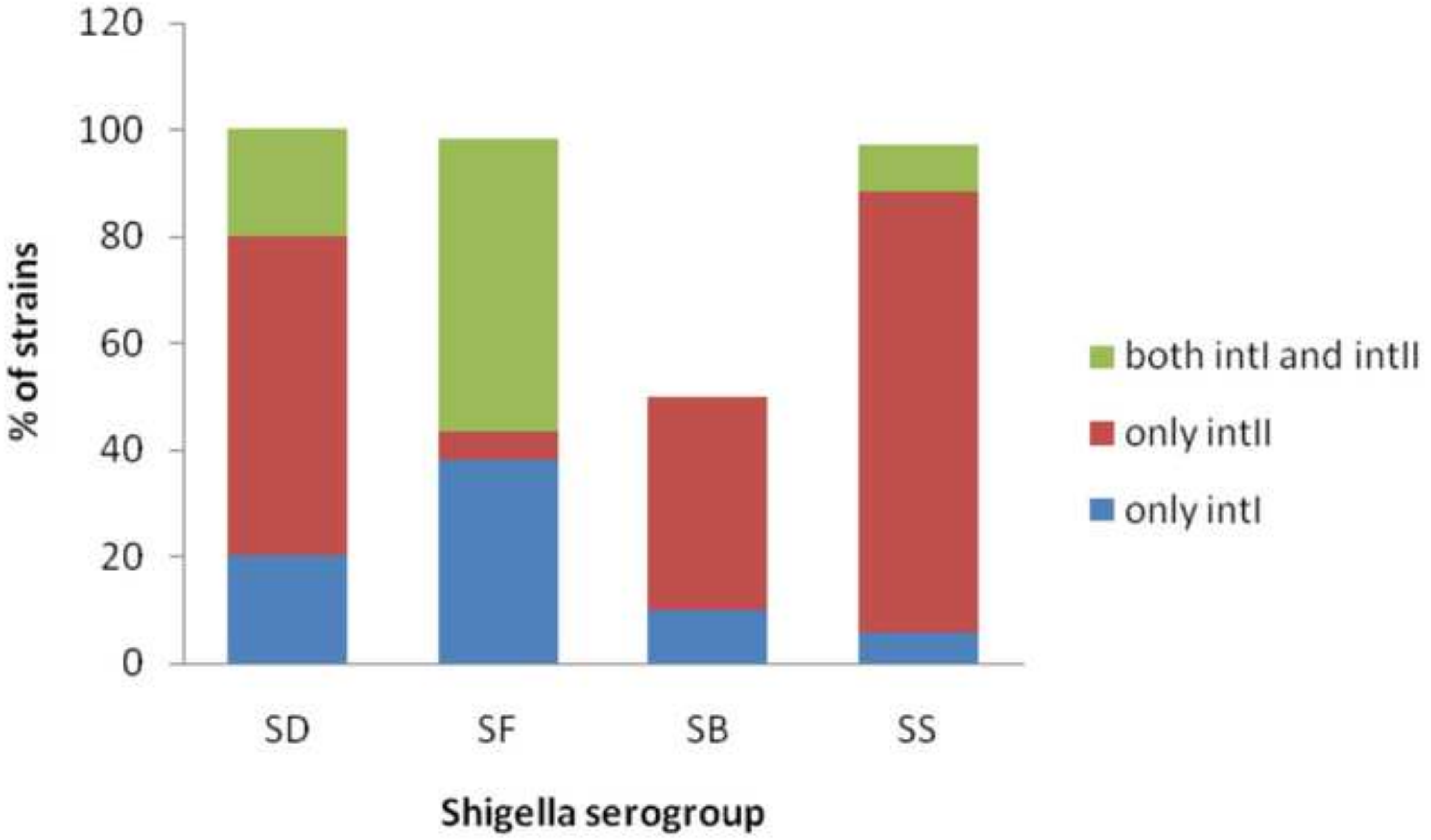

Figure 6

Click here to access/download;Figure;Fig 6.tif

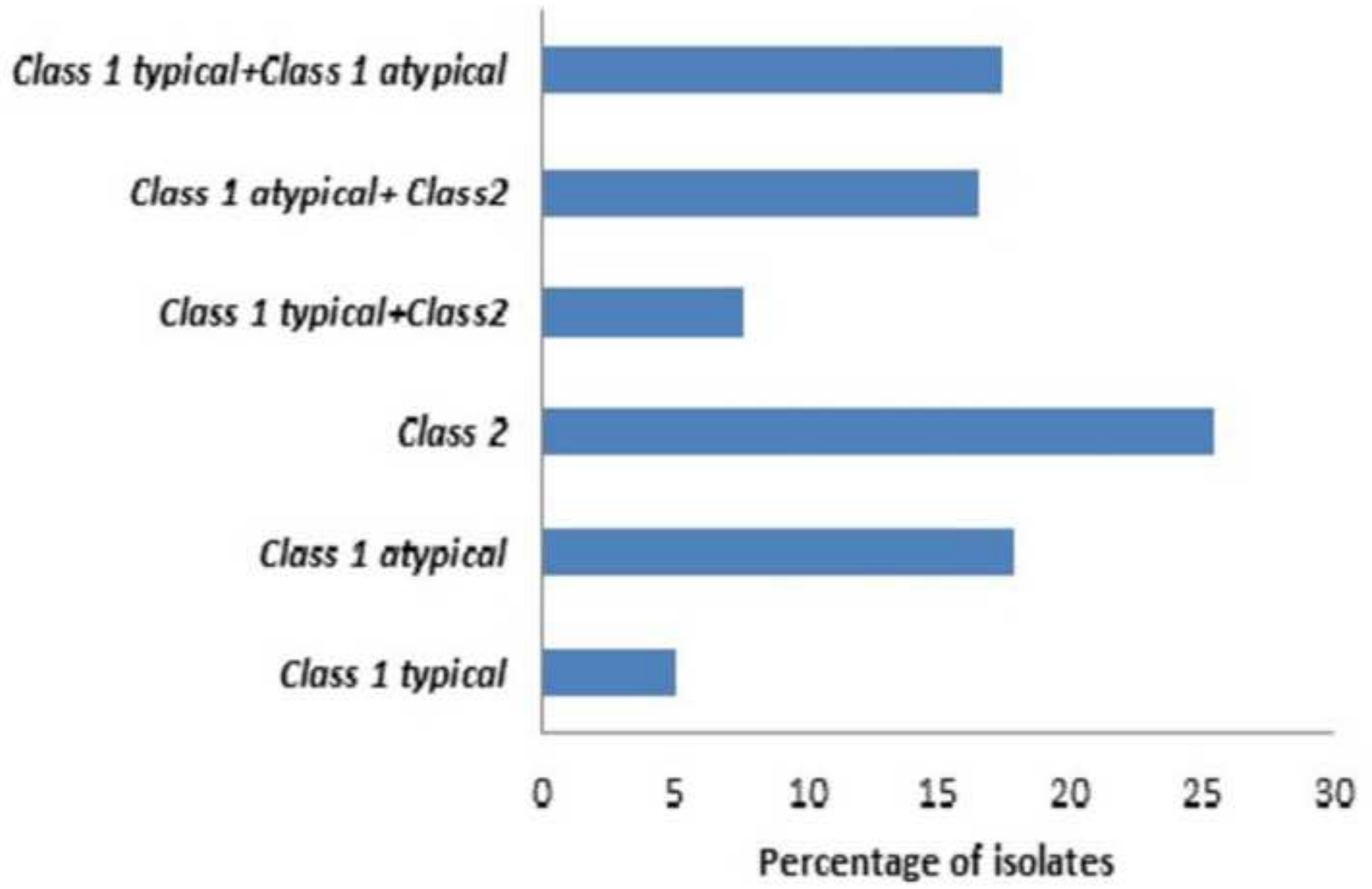

 Click here to access/download;Figure;Fig 7.tif

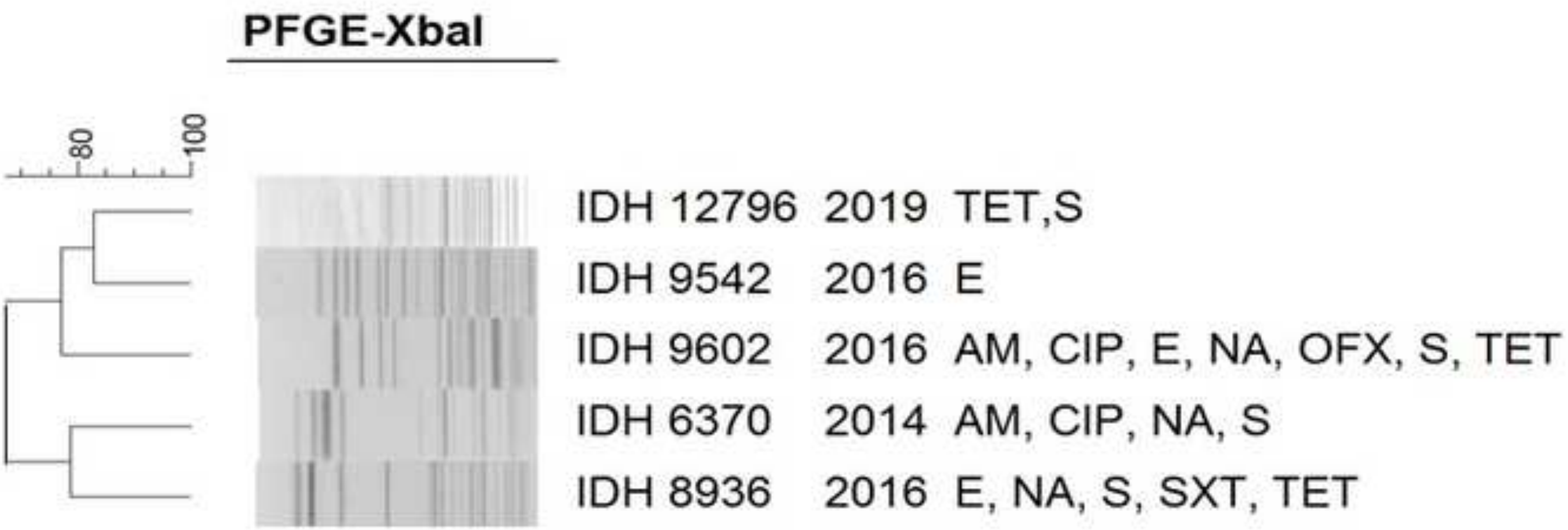

Figure 8

Click here to access/download;Figure;Fig 8.tif

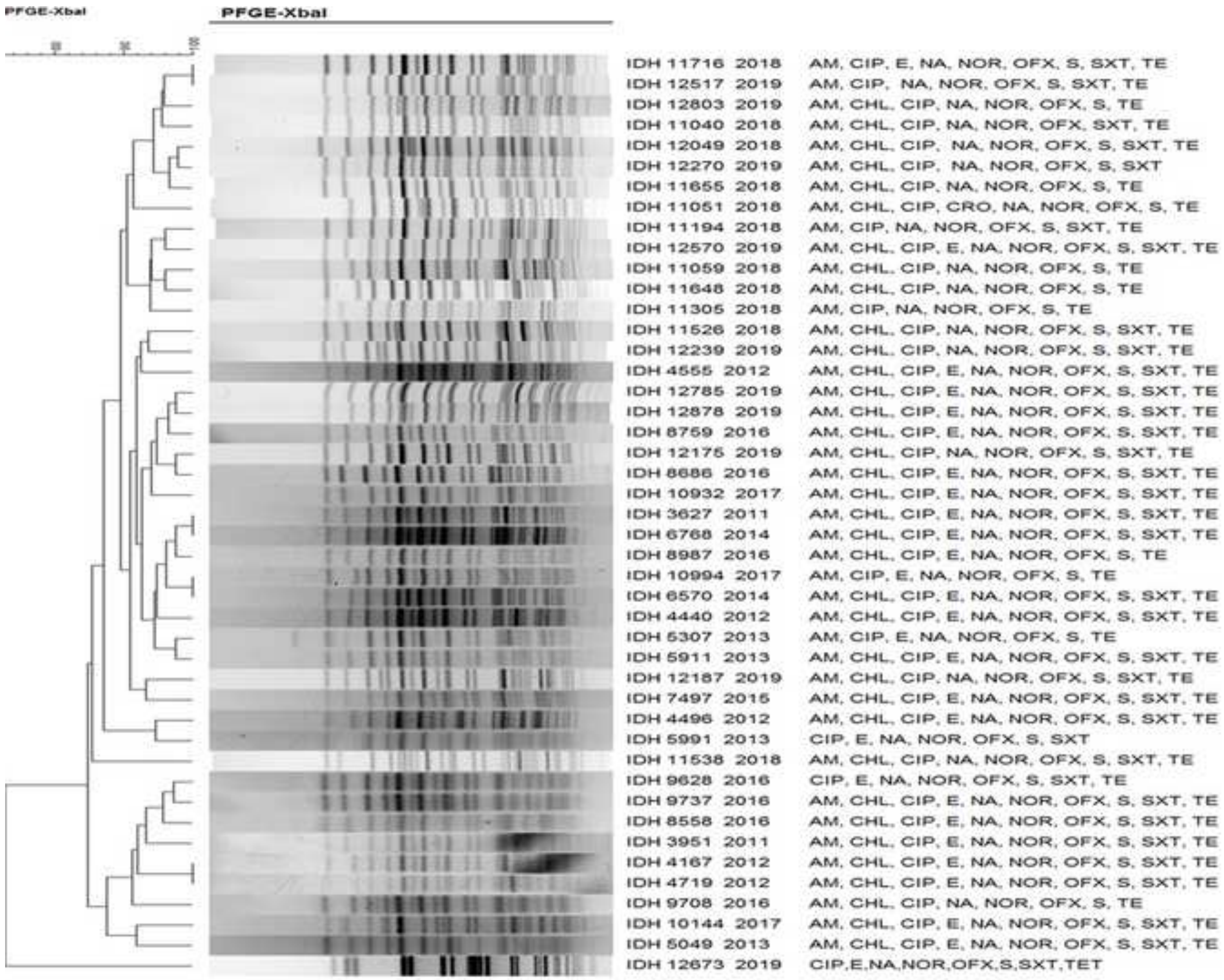

Figure 9

Click here to access/download;Figure;Fig 9.tif

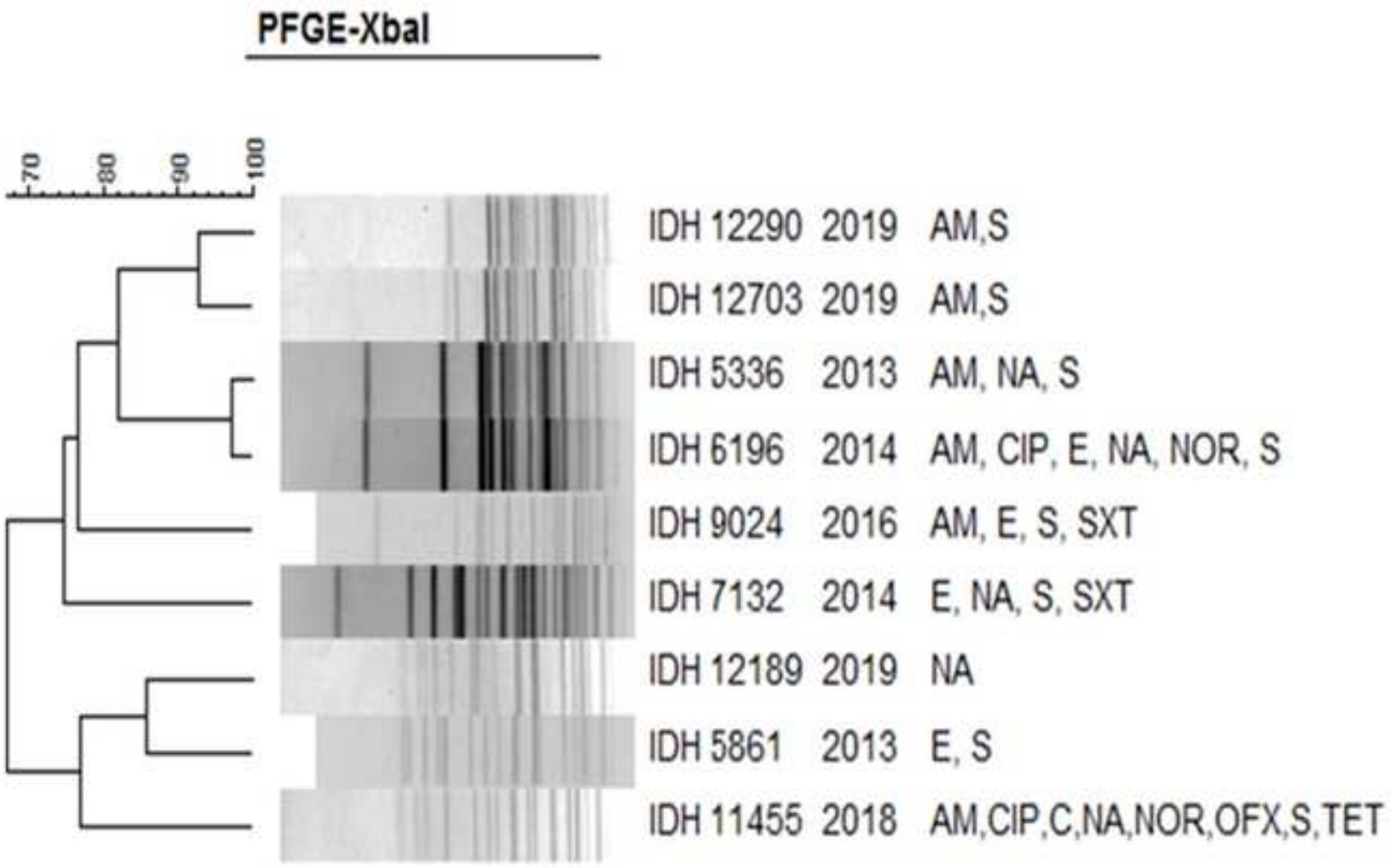

Figure 10

Click here to access/download;Figure;Fig 10.tif

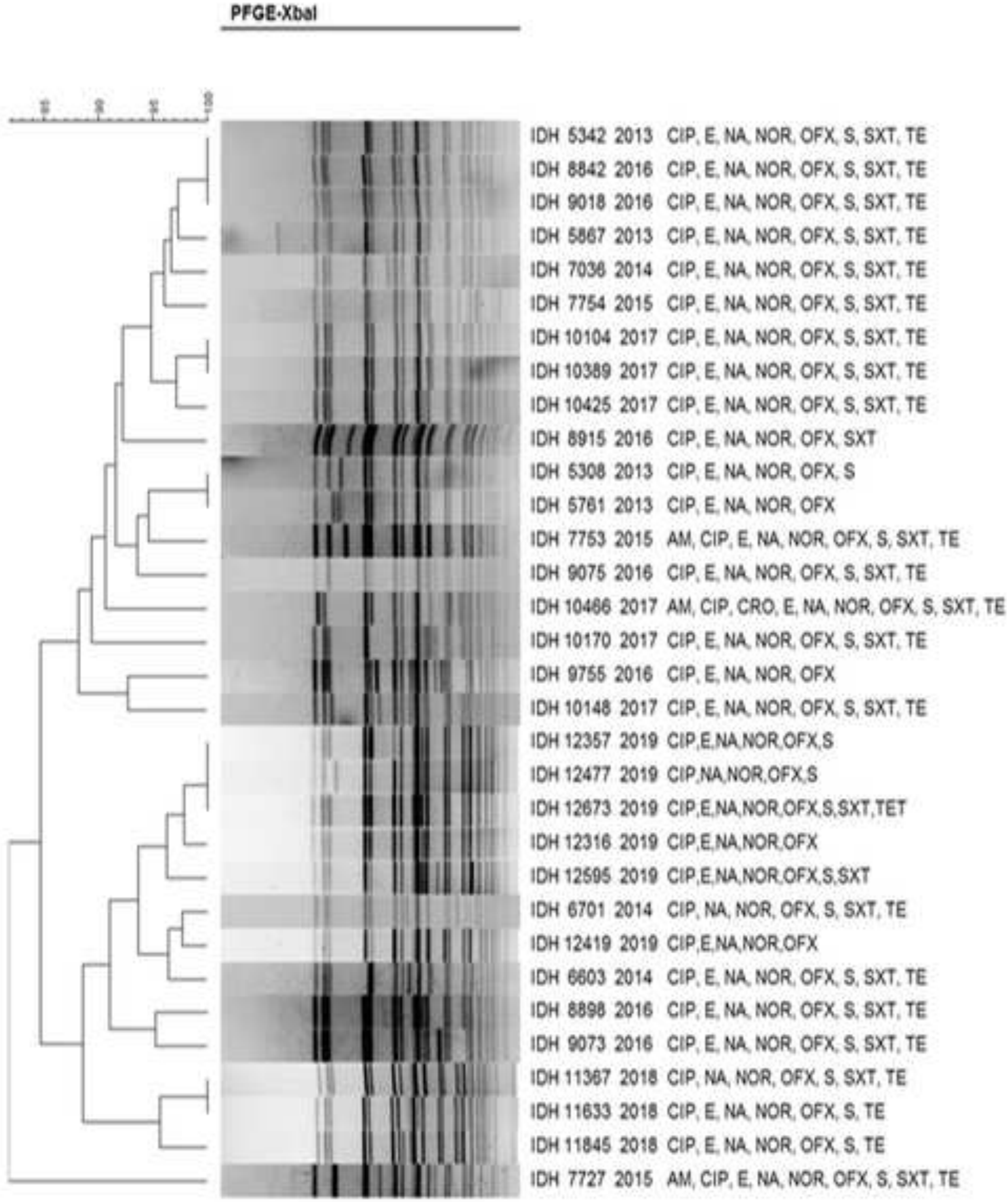

Supporting Information fig1

Click here to access/download
Supporting Information
S1_Fig.tif

Supporting Information fig2

Click here to access/download
Supporting Information
S2_Fig.tif

Supporting Information fig3

Click here to access/download
Supporting Information
S3_Fig.tif

Supporting Information fig4

Click here to access/download
Supporting Information
S4_Fig.tif

Supporting Information fig5

Click here to access/download
Supporting Information
S5_Fig.tif

Supporting Information table 1

Click here to access/download
Supporting Information
S1_Table.docx

Supporting Information table 2

Click here to access/download
Supporting Information
S2_Table.docx
