# Supplementary material for: Prevalence and changing antimicrobial resistance profiles of Shigella spp. isolated from diarrheal patients in Kolkata during 2011–2019
Source: PLoS Negl Trop Dis. 2024 Feb 20;18(2):e0011964. doi: 10.1371/journal.pntd.0011964 (PMC10906866; doi:10.1371/journal.pntd.0011964)
Supplement: Attachment — Submitted filename: PLOsNTD resubmission cover letter.docx [file pntd.0011964.s009.docx]

Dr. Asish K. Mukhopadhyay	January 15, 2024

Division of Bacteriology

ICMR-National Institute of Cholera and Enteric Diseases,

P 33, CIT Road, Scheme XM, Beliaghata

Kolkata 700010

India

E-mail: asish_mukhopadhyay@yahoo.com

FAX: 91-33-2370-5066

To

Dr. Shaden Kamhawi and Dr. Paul Brindley

Editors-in-Chief

PLOS Neglected Tropical Diseases

Dear Sir,

I am resubmitting our manuscript entitled “**Prevalence and changing antimicrobial resistance profiles of *Shigella* spp*.* isolated from diarrheal patients in Kolkata during 2011-2019**” along with 10 figures and 7 supplementary files as attachments.

Previously, this manuscript was submitted to this journal on 29th September, 2023 (manuscript no. PNTD-D-23-01227) and **Dr. Sivanantham Krishnamoorthi and Dr. Stuart Blacksell** have been assigned as the Guest Editor and Section Editor, respectively of this manuscript. Our manuscript has been reviewed by members of the editorial board and several independent reviewers. On 28^th^ December, 2023 based on the peer-review process, the editorial board offered the decision to accept the manuscript after revising it according to the reviewer’s comments.

We have revised the manuscript thoroughly according to the reviewer’s comments with the addition of suggested information, modified tables, and prepared a point by point response to the reviewer’s comments. The details are followed herewith for your kind perusal.

I confirmed that all the authors contributed significantly to this work and have checked and approved the manuscript for resubmission.

Thank You,

Yours faithfully,

Dr. Asish K. Mukhopadhyay

**Responses to the Reviewers’ comments**

We are grateful to both the editor and reviewers for their insightful comments, which we have seriously considered in the revised manuscript.

**Reviewer #1: No changes suggested**

The authors are thankful to the reviewer for his contribution to this peer-reviewed process.

**Reviewer #2: Minor Revision**

1. **Line 110: Mention the references of studies that have been made across the world to track the epidemiology and antimicrobial susceptibility patterns of *Shigella* spp.**

**Answer:** Thank you for the suggestions. The mentioned references of studies that have been made across the world to track the epidemiology and antimicrobial susceptibility patterns of *Shigella* spp. has been added in the revised manuscript (Line: 110).

2. **Mention the stool macroscopic characteristics (bloody diarrhoea/ watery diarrhoea).**

**Answer:** The following sentence of stool macroscopic characteristics has been added in the revised manuscript (Line: 216).

“The macroscopic characteristics of stools related to Shigellosis have different characteristics like, majority of the infected stools had muciod consistency (48%), 31% was bloody diarrheal stool, 12% was watery diarrhoeal stool, whereas 4% and 5% were semisolid stool and swab, respectively”.

3. **Whether all *Shigella spp.* were typable or few non-typeable strains were there.**

**Answer:** Thank you for the query.

Among 407 *S. flexneri* strains, 13 strains were designated as *S. flexneri* UT those were non-typeable strains as they only agglutinated with polyvalent type B antiserum; further serotyping of these isolates could not be completed with commercially available *Shigella* antisera. This was already mentioned in the manuscript line number 258.

4. **Please mention why during AMR gene detection for β-lactamase genes blaOXA and blaTEM was choosed and not CTX-M?**

**Answer:** The authors acknowledge the concern of the reviewer. The authors want to mention that CTX-M screening was also done during this study, among 32 third generation cephalosporins-resistant isolates, only 12 (37.5%) showed the presence of the bla_CTX-M_ genes. As we have considered CTX-M gene related to ESBL isolates,so the data was not mentioned previously. Now the data has been added to the revised manuscript at line number 335.

5. **Mention in discussion regarding the gyrB and parC prevalence in comparison to your findings both in India and globally.**

**Answer:** Authors thanked the reviewer for this valuable suggestion.

There is only one mutation reported in China about *gyrB* gene at 517th position. In our study we didn’t notice any mutation in *gyrB*. Whereas, for *parC* gene we have only found mutations in 80^th^ position of *parC*, there are different numbers of mutations reported around the world, among them five types of mutation in *parC* gene is reported from India previously including 80^th^ position like our study. Discussion regarding the *gyrB* and *parC* prevalence in comparison to our finding both India and globally has been added in the discussion part with proper reference (Line: 424) in the revised manuscript.

6. **Grammatical corrections needed in the introduction and discussion part.**

**Answer:** Thank you for these suggestions.

The manuscript was checked thoroughly and the grammatical mistakes are corrected especially in the introduction and discussion part.

7. **Conclusion and limitation of the study, if any to be mentioned.**

**Answer:** The conclusion (Line 460) and limitation (Line 444) of the study have been added in the revised manuscript.

8. **Study design, statistical analysis method and ethical approval to be mentioned.**

**Answer:** The authors are thankful to the editor for this suggestion. The study design (Line:123), Statistical analysis (Line: 207) and Ethical approval (Line: 117) have been added to the revised manuscript.

9. **Ethical approval to be mentioned.**

**Answer:** The authors added Ethical approval (Line: 117)to the revised manuscript.

**Reviewer #3: A few minor edits are needed, as indicated in the reviewed file**.

1. **The corrections in the authors summary part (reviewed manuscript Line:54)**

**Answer:** Thank you for suggesting the correction.

2. **Ethical approval statement shall include the approval number (reviewed manuscript Line: 126)**

**Answer:** The authors are thankful for pointing out this issue.

The ethical approval statement has been added to the revised manuscript. (Line:117)

3. **The exact CLSI document can be cited like M02 (reviewed manuscript Line: 133).**

**Answer:** All the authors are grateful for pointing out this topic.

The exact CLSI document M100, 30^th^ edition has been mentioned and cited appropriately (Line: 141).

4. **The exact CLSI document can be cited like M02 and M100 for interpretation (reviewed manuscript Line:139)**

**Answer:** All the authors are grateful for pointing out about this topic.

The exact CLSI document M100, 30^th^ edition has been mentioned and cited appropriately (Line: 151).

5. **Table to adjusted to fit in (reviewed manuscript Line: 216).**

**Answer:** Thanks for the suggestion. Table1 has been adjusted to fit in size in the revised manuscript.

6. **Data availability and ethical approval statement to be added (reviewed manuscript Line 429).**

**Answer:** Authors are thankful for the valuable suggestion the data availability was actually mentioned during the submission of the manuscript to the submission system and mentioned as “All relevant data are within the paper and its Supporting Information files. The sequences submitted in the GenBank are available with accession number PP196524 to PP196533”. It was mentioned in the system that “This statement will be typeset if the manuscript is accepted for publication”.

As mentioned earlier, ethical approval statement has been added to the revised manuscript.
